# Dynamics of the Global Wheat Trade Network and Resilience to Shocks

**DOI:** 10.1038/s41598-017-07202-y

**Published:** 2017-08-03

**Authors:** Kathyrn R. Fair, Chris T. Bauch, Madhur Anand

**Affiliations:** 1 University of Waterloo, Department of Applied Mathematics, Waterloo, N2L 3G1 Canada; 2 University of Guelph, School of Environmental Sciences, Guelph, N1G 2W1 Canada

**Keywords:** Ecological modelling, Applied mathematics

## Abstract

Agri-food trade networks are increasingly vital to human well-being in a globalising world. Models can help us gain insights into trade network dynamics and predict how they might respond to future disturbances such as extreme weather events. Here we develop a preferential attachment (PA) network model of the global wheat trade network. We find that the PA model can replicate the time evolution of crucial wheat trade network metrics from 1986 to 2011. We use the calibrated PA model to predict the response of wheat trade network metrics to shocks of differing length and severity, including both attacks (outward edge removal on high degree nodes) and errors (outward edge removal on randomly selected nodes). We predict that the network will become less vulnerable to attacks but will continue to exhibit low resilience until 2050. Even short-term shocks strongly increase link diversity and cause long-term structural changes that influence the network’s response to subsequent shocks. Attacks have a greater impact than errors. However, with repeated attacks, each attack has a lesser impact than the previous attack. We conclude that dynamic models of multi-annual, commodity-specific networks should be further developed to gain insight into possible futures of global agri-food trade networks.

## Introduction

As nations become more interconnected in the era of globalisation, trade networks play an increasingly significant role in the well-being of nation states. The ways in which countries select trading partners; the global impact of local economic crises due to globalisation; and how country-level characteristics are affected by network metrics can be explored by analyzing these trade networks^1^. A significant amount of research has characterized trade networks and described how they change over time. However, a crucial subset of these networks–trade in agri-food commodities–has not been explored in as much depth. Trade in agri-food products will become more crucial as global population growth, urbanization, and shifting consumption patterns decrease land resource availability^2^. Global food exports began increasing exponentially after the 1960s and are growing more rapidly than food production^3^. This upswing in exports, along with the 50% increase in food demand predicted to occur by 2030, indicates that agri-food trade will only increase in political and economic importance^4^.

Potential risks to the agri-food network are abundant, with more emerging due to a variety of factors. In trade networks shocks generally manifest as the sudden inability of countries to export due to a negative supply shock that alters network structure^5–8^. A myriad of triggers can cause these shocks. The globalisation of agri-food trade has led to the threat of contaminants spreading across international borders, forcing countries to close their borders to trade and affecting billions of people^9–11^. Reductions in availability and quality of cereal crops due to extreme weather conditions impacting major agricultural producers will only be worsened by climate change^12–14^. Food crops being utilised in fuel production and higher demand for meat in low-income nations results in increased pressure on global stocks of vegetable and cereal crops^12^. Other shocks that could impact global food supply include agro-terrorism, crop pests, and epidemics^13, 15–18^. During food shortages countries often cease exporting agri-food commodities^13, 19–29^. For example, as a result of the global food crisis in 2008 trade restrictions were imposed by 6 of the top 17 wheat exporters and 4 of the top 9 rice exporters^13, 20^. These export restrictions caused increased global prices and led to other countries imposing export restrictions, resulting in even higher global prices in what has been dubbed a “multiplier effect”^30^. As network connectivity increases these disturbances could severely impact low-income countries which depend greatly on imports of staple foods during shortages and are heavily burdened by the resulting price shocks^13, 31^.

Previous network analyses of the agri-food trade network as a whole^3, 32^: the virtual water trade network^5, 6, 33–36^; and commodity-specific trade networks^7, 8, 11–13, 37^ have been undertaken with some consideration of shocks in relation to food security^3, 5–8, 12, 13, 38^. These studies have led to conflicting views on how globalisation has impacted global food security in these networks. Several reference the heterogeneous degree distributions of these networks or their clustering as sources of vulnerability^3, 11, 32, 33, 35, 38^, while others state that the globalisation of trade has had little negative effect on global food security^6^. Network vulnerability, measured using the magnitude of damage to network structure resulting from shocks, is one aspect of network resilience. Definitions of resilience encompass not only the robustness of a network to damage resulting from shocks but also the speed at which it recovers from shocks^39^. Further research, especially regarding the resilience of trade networks for globally important staple foods, could provide useful insights and guide the development of food security policy.

While static or descriptive analyses of network structures can be helpful, models that examine the network formation processes may be useful for understanding mechanisms that determine network structure^40^. By modelling a trade network it becomes possible to perform experiments *in silco* to gain insights into network dynamics, both in terms of future growth and response to shocks that might be experienced under different possible future scenarios. This can help ensure that policy is pro-active instead of reactive^13^.

A review of the literature reveals that most trade network models focus either on network formation or on simulating shocks. Network formation models generate a network according to growth rules and then treat the final network as a static, single-year “snapshot” of the empirical network with different model parametrisations for each snapshot. Shock simulation models generally do not include growth mechanisms, meaning that the interaction between network growth and response to shocks is not considered^5, 8, 13^. These approaches do not permit an examination of the concurrent multi-year effects of temporal network evolution and shocks on these networks–areas we propose to explore here.

Additionally, network formation models have been formulated only the network of all globally traded goods (WTN: the world trade network)^38, 41–45^ and the virtual water trade network (VWTN)^46^, to our knowledge. Because networks for individual commodities often have structural differences compared to the entire WTN, models describing their evolution could differ in important ways from those created to represent the entire WTN. Therefore models of individual commodity networks should be further studied since they may yield unique insights^37^.

To gain insight into these complex and important networks, an understanding of how they form and grow is critical. Two main theories as to how trade networks evolve over time have been proposed. In an analysis of “rich get richer” preferential attachment (PA) models and homophily models of political and economic networks, Maoz found that partnerships in the WTN form according to preferential attachment based on the total degree centrality of nodes^40^. Preferential attachment captures the fact that nation states that already have a high node degree or centrality are often more desirable as potential trade partners^40, 47^. Alternatively, Garlaschelli and Loffredo assert that the mechanism driving the growth of the WTN is a “good get richer” system, represented by a model based on the hidden variable hypothesis (HVH). In this case, each node has an intrinsic fitness that will impact the probability of connection. They identified the hidden variable (fitness) for the WTN as annual GDP and used this to model the growth of the WTN in specific years^41, 42^.

While the HVH requires knowledge of economic data such as annual GDP, the PA model can be implemented without such data. Hence, its relative simplicity and fewer data requirements, together with the current knowledge gap on applying PA models for such problems, make it an attractive prospect for modelling dynamics of single commodity agri-food networks. Based on this review of the literature, we formulated 2 questions to motivate our work. First, can a preferential attachment model describe the growth of a trade network for a specific commodity over many years? Second, if a PA model can describe such a network, how does that model predict the network will respond to shocks? Our corresponding objectives were twofold: to fit a PA model for a commodity-specific network using empirical data, and to use that model to predict the response of that network to shocks.

To this end, we constructed a dynamic model of the global wheat trade network that builds on previous work by considering the temporal evolution of the network and how shocks impact it over time. The PA model was calibrated to describe the time evolution of the empirical network. We carried out a vulnerability analysis of both empirical and model networks to ascertain potential weaknesses, and to determine whether the network is evolving over time to become more or less resilient in the face of shocks.

**Figure 1:**
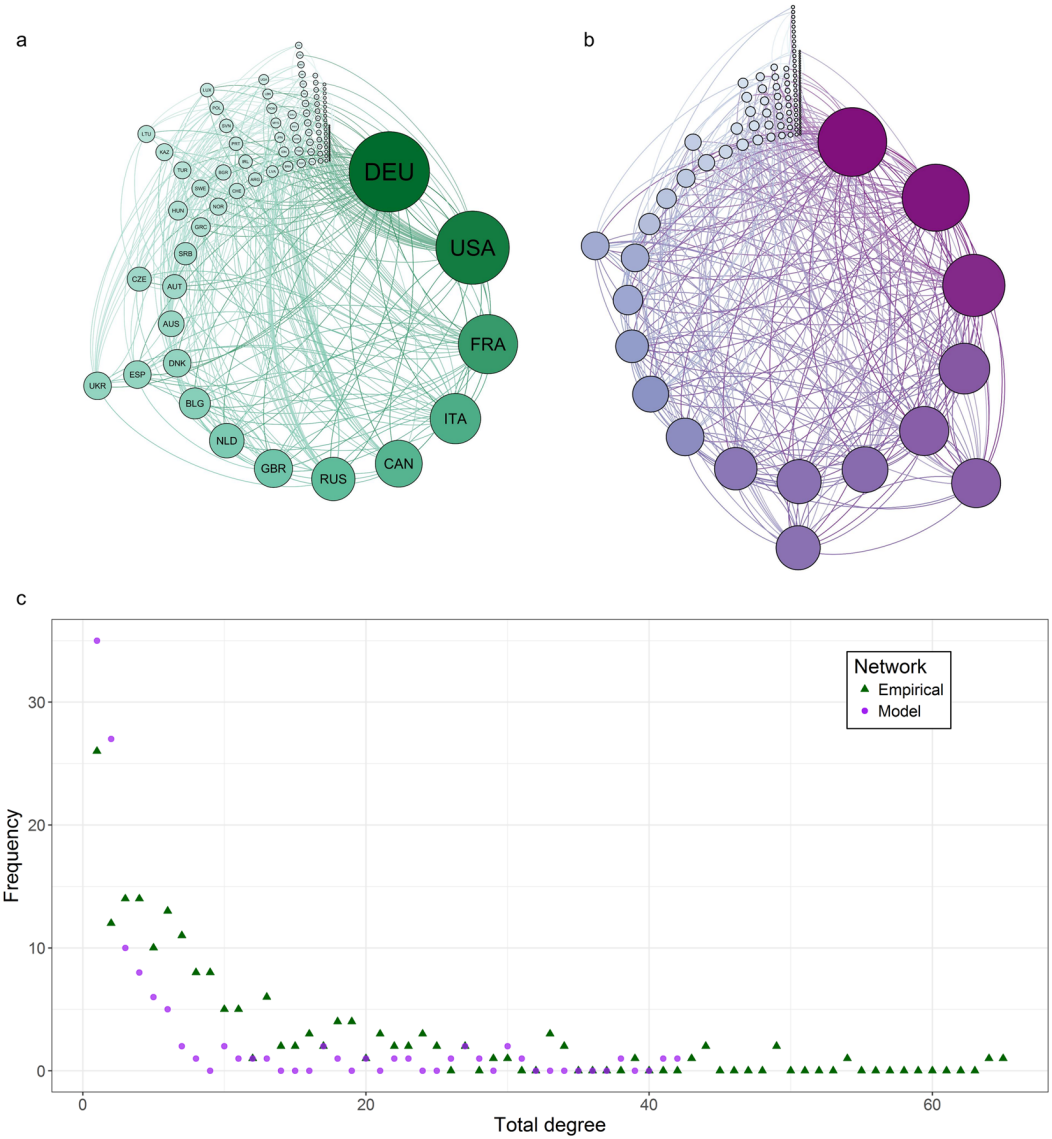
Comparison of empirical and model continuous wheat trade networks, 2013. (**a**) Empirical network. (**b**) Exemplar model network. (**c**) Comparison of degree distributions for empirical and exemplar model networks. All sub-figures are for the network at the end of the year 2013. For (**a**) and (**b**), nodes are arranged clockwise, and in size and colour, by total degree. In (**a**) node labels correspond to the ISO 3166–1 alpha-3 codes for the countries they represent.

The global wheat trade network (Fig. 1a) was chosen for analysis due to its global importance. From 1986–2010 wheat was the highest traded agri-food commodity by volume, and in the top 10% of agri-food commodities both in regard to number of countries trading and the number of trades^48^. Shocks impacting this network have had significant impacts on global food security, most recently during the 2007–08 world food crisis and in 2010–11 when several major producers imposed export restrictions^13, 21–25, 27, 29, 49^. An additional cause for concern is that shocks impacting global agri-food trade, and the wheat trade network specifically, are expected to occur more frequently as we move further into the 21st century^50, 51^.

## Methods

### Definition of Empirical Network

We began by defining the network we sought to model as a subset of the entire global wheat trade network. To define a network we followed an approach similar to the existing practice of creating a “backbone” network. These networks include only those edges corresponding to the largest trades by volume and together accounting for 80% of total trade volume. This method is used to simplify analysis of trade networks while also retaining salient aspects of network structure^3, 13, 33, 46, 52^.

Previous research defines a backbone network for static networks and often over a single year only. In contrast, we defined a “continuous wheat trade network” by including only edges where trade was sustained over at least 3 years (see Supplementary Information). We therefore simplified the model by avoiding having to determine when and how edges should be deleted. Also, using 3 years instead of 1 year increases the chance that our network reflects the long-term features of network structure. We also assume that countries connected by a trade link persisting over multiple years have a higher probability of being impacted by a shock than countries that are only infrequently part of the network. Thus, continuous trade networks are of interest when modelling long-term dynamics and the long-term impact of shocks on the biggest traders.

While our network is not defined in the same way as a conventional backbone network, it accounts for 66% of total trade volume despite containing only 30% of the trades, on average (see Supplementary Fig. S7). By 2013, there were 108 countries engaged in 363 continuous trade partnerships in this network^48^. The correlation between a country’s total degree in the continuous trade network and its total trade volume in the empirical network is positive and significant at the 5% level (see Supplementary Fig. S8). This suggests that countries that are important to our continuous trade network are also important in a conventionally-defined backbone network. The characteristics of the empirical network are further described in the last subsection of Methods.

### Overview of Model Network

We developed a preferential attachment (PA) model in order to mechanistically describe the empirical network. The PA theory of trade network formation operates on the premise that well-connected countries (namely, countries with high total degree centrality) are more appealing to prospective trading partners^40, 47^. As already noted in the Introduction, PA theory has not been applied to trade networks for specific commodities, to our knowledge. Additionally, using a PA model does not require annual GDP as input data, in contrast to the Hidden Variable Hypothesis (HVH) model, and therefore offers the possibility of a more parsimonious theoretical model.

However, to create our PA model, we built on certain aspects of the HVH model of trade network formation proposed by Garlaschelli *et al*. (see following subsections for details)^41, 42^. For a PA model to describe a commodity network, the number of nodes in the network must grow over time, and new nodes must initiate trades with highly-connected pre-existing nodes (i.e. the network must be disassortative). These characteristics are observed in the continuous wheat trade network we wish to model (Supplementary Fig. S4).

Our model of the wheat trade network (Fig. 1) contains *m* directed, unweighted edges and is represented by an *N* × *N* matrix *A* where *N* is the total number of countries in the world. The (*i*, *j*)^*th*^ element of the matrix contains information on import from country *j* to country *i* (*A*(*i*, *j*) = 1 if *i* imports from *j*, and *A*(*i*, *j*) = 0 otherwise). The way in which trade partnerships are formed to populate this matrix is discussed in Trade Probability Calculations. The rate of network growth, in terms of number of edges, was estimated by extrapolating 26 years of data from the United Nations Food and Agriculture Organization (FAO) (see Supplementary Information)^48^. As we considered a directed network, imports and exports between countries *i* and *j* were represented as separate partnerships: if these countries engage in reciprocal trade, they will possess 2 partnerships. All simulations were run in Matlab R2014b^53^. Network analysis was conducted using the igraph package (version 1.0.1)^54^ for Rstudio (version 3.2.2)^55^. Network visualization was conducted using Gephi (version 0.8.2)^56^.

### Trade Probability Calculations in Network Model

The probability of a trade (import or export) occurring depends on the fitness of both countries involved in the potential trade. For the PA model, a country’s trade fitness depends on their total degree centrality^40, 47^. We defined the probability *P*_*t*_[*x*_*i*_(*m*), *x*_*j*_(*m*)] of a trade between countries *i* and *j* when there are *m* directed edges in the network as

1 $${P}_{t}[{x}_{i}(m),{x}_{j}(m)]=\frac{\alpha {x}_{i}(m){x}_{j}(m)+\varepsilon }{1+\beta {x}_{i}(m){x}_{j}(m)},$$

where *x*_*i*_(*m*) and *x*_*j*_(*m*) are the fitness for country *i* and country *j* when there are *m* directed edges in the network, and $\varepsilon \ll 1$ is some small probability of the formation of an edge between countries *i* and *j* when one or both has zero fitness (i.e. one or both are not yet engaged in any trade partnerships). *α* and *β* are free parameters that we fit to match characteristics of the empirical network (see Comparison of Empirical and Model Networks)^42^. *α* scales the overall probability of connection whereas *β* controls how strongly the probability of connection depends on fitness. For example, a very small *β*-value ($\beta \ll 1$) would allow approximating equation (1) by ${P}_{t}[{x}_{i}(m),{x}_{j}(m)]\approx \alpha {x}_{i}(m){x}_{j}(m)+\varepsilon$, and the product ${x}_{i}(m){x}_{j}(m)$ would have a large impact on the overall probability.

It could be argued that a country with high export fitness would tend not to have high import fitness. In this case, separate probabilities of import and export should be calculated based on import and export fitness values for each country. However, we do not believe this to be necessary. We examined the top 20 wheat importing and exporting countries by year for 1961–2011, determining that 25% of countries were in the top 20 for both import and export volume each year^48^. Similar results have been found for the maize network from 2000–2009^11^. This overlap between the largest importers and exporters suggests that there is considerable overlap between import and export fitness.

We defined the fitness of country *i*, when there are *m* directed edges in the network, as

2 $${x}_{i}(m)=\frac{{l}_{i}(m)}{m},$$

where *l*_*i*_(*m*) is number of edges connected to country *i* when there are *m* directed edges in the network (the country’s total degree). The degree of each node (country) is recalculated every time a new edge is added to the network. Thus, a country’s fitness is dictated by the fraction of total trades it is involved in, and is a unit-less quantity. Both the trade probability and fitness equations are adapted from previous research^42^.

### Model Network Formation

At each time-step a series of events occur that may lead to a new edge being added to the network (see Supplementary Fig. S9). The likelihood of a new edge being formed increases with the fitness values of the countries involved in the potential trade. Our model permits reciprocal trades, where a country both imports and exports the same good with the same partner country. We allow for this as our analysis shows reciprocal trade within the continuous wheat trade network (see Supplementary Fig. S4), and Shutters and Muneepeerakul note the existence of reciprocal trades within agricultural trade networks^57^. However, self-loops, where a country attempts to trade with itself, are excluded.

Initial attempts to emulate the observed characteristics of the empirical network, as described in Comparison of Empirical and Model Networks, revealed that our model networks were not as disassortative as the empirical network. To address this, we introduced a step of rewiring the network after the addition of each new edge using the Maslov-Sneppen rewiring algorithm (MSRA), which has been shown to increase disassortativity. This algorithm ensures that node degree is not impacted by rewiring and edges are uniquely defined^58, 59^. Our rewiring led to a better fit of model to empirical network in terms of assortativity (see Supplementary Fig. S5)^59^. The number of attempts to rewire the network (*R*(*m*)) decays exponentially as the number of directed edges (*m*) in the network increases:

3 $$R(m)=C{e}^{-\lambda m}.$$

This functional form was chosen as it results in a good fit to the increases in assortativity over time in the empirical network. Rewiring acts as a random assignment of trade partnerships for countries that are already engaging in trade. The good fit to the empirical network provided by an exponentially decaying number of rewiring attempts indicates that as the network grows, the PA mechanism becomes increasingly dominant over these random partnership assignments.

### Comparison of Model and Empirical Networks

We utilised a grid sweep to calibrate values of *α*, *β*, and *ε* from equation (1) as well as *C* and *λ* from equation (3). This generated approximately 5000 possible parameter sets (see Supplementary Information). Previous analysis has calculated *α* and *β* for an HVH model of the WTN in individual years^42^. However, we wanted a single set of parameters that would replicate the characteristics of the empirical wheat trade network over multiple years. Thus, we carried out a parameter fitting instead of calculating parameter values corresponding to specific years. The parameter fitting sought a parameter set with a good fit to the number of nodes, reciprocity, and assortativity coefficient of the empirical network for 1986–2011. The number of nodes and reciprocity were chosen as fitting metrics because the functional forms for trade probabilities taken from Garlaschelli *et al*. are related to the number of nodes and network reciprocity^41, 42^. The requirement on assortativity arises because an assortative network responds differently to shocks than a disassortative network^60–62^.

To determine the best parameter sets, 25 networks were generated for each parameter set and network metrics were averaged over these networks. The mean squared error (MSE) from 1986–2011 between the model and empirical networks for assortativity, number of nodes and reciprocity was calculated for each parameter set. For each metric the MSE values were normalized, and then a combined normalized MSE over these metrics was generated. Parameter sets were ranked according to their combined normalized MSE, and the 100 “best” sets with the lowest MSE were used for all subsequent analysis.

We focused on a subset of network metrics to simplify the analysis of predicted dynamics of the model network and its response to shocks under different scenarios. To pick these metrics we examined a wide range of metrics for empirical agri-food commodity trade networks^48^. This analysis revealed strong correlations between many metrics, allowing us to reduce the number of measures needed to describe a network fully (see Supplementary Information). Average path length, assortativity, and average clustering coefficient were thereby chosen as network metrics. The average path length is the average of the all the shortest paths between pairs of nodes; assortativity, by degree, is a measure of the extent to which nodes of a similar degree tend to connect to each other; the average clustering coefficient describes the degree to which a node’s neighbours are themselves linked, averaged over the entire network^63^.

We also included the sizes of the giant strong component (GSC) and giant weak component (GWC) because of their relevance to network vulnerability analysis^15–18, 64–70^. The GSC and GWC are defined as the largest strongly- and weakly- connected components within the network, respectively^17^. The size of a component – a collection of connected nodes – is given by the number of nodes it contains^15, 16, 18^. In a directed network, a strongly-connected component contains nodes which can all reach each other. A weakly-connected component contains all nodes in the strongly connected component, as well as any nodes that could reach all other nodes in the component if the network were undirected^17^.

In addition to metrics of the vulnerability of networks to shocks we included 3 others that are thought to impact network resilience: density, symmetry, and heterogeneity (the latter 2 in terms of node in- and out-degree)^71^. Network density is the fraction of the maximum possible number of links that are present in the network^72^. Symmetry, in terms of node in- and out-degree, describes the extent to which nodes with a high in-degree also tend to have high out-degree. Degree heterogeneity measures the heterogeneity in the in- and out-degree distributions of the network^71^. Network analysis often includes a consideration of whether the degree distribution of the network follows a power-law. The presence of a power-law distribution can can be determined using the Kolmogorov-Smirnov (KS) statistic. However, the KS statistic is not accurate for networks containing small numbers of nodes (approximately 100 or fewer nodes)^73^. Thus, the KS statistic was only used to determine the shape of the degree distribution in networks containing more than 100 nodes. For all other metrics, a network was generated for each of the 100 best parameter sets, and metrics were averaged across these 100 networks for comparison to the empirical network.

### Shock Simulation

To ascertain the resilience of the wheat trade network and its response to shocks we simulated shocks to the model networks and compared the effects of different types of shocks on model network structure. Throughout the shock analysis 1 network was generated for each of the 100 best parameter sets, and metrics were averaged across these 100 networks. Shocks to agri-food trade networks generally result in countries imposing export restrictions, while continuing to import^13, 21–27, 29, 38, 49, 74^. Thus, we implemented shocks that result in nodes having their outgoing (export) edges removed^13, 38, 74^.

Studies have considered 2 types of shocks to a network: errors and attacks^13, 15, 65, 75, 76^. In the case of an error we removed the outgoing edges of randomly selected nodes to test the network’s error tolerance^65, 75, 76^. For an attack, nodes with the highest connectivity are targeted, as these are assumed to be the most important nodes in the network^65, 77^. We defined connectivity in terms of total degree centrality, meaning the nodes with the most trade partners were targeted for outward edge removal. The total degree metric was not recalculated between removals; we used a simultaneous attack that assumes all countries targeted would be impacted at roughly the same time, as this reduces computational time^64, 77^. In addition to single shocks, we experimented with introducing multiple shocks to the networks.

We also explored variations. Attacks with removals based on out-degree centrality (removal of the countries with the most export links) were simulated^76^. A sequential attack was considered, as it may not be realistic to assume that cessation of exports in multiple countries occurs simultaneously, and 1 country’s change in trade status will impact the centrality of other countries in the network^77^. Details of how shocks were implemented appear in Supplementary Information.

We use empirical data to determine approximate ranges for the number of countries affected by a shock (1–15 countries) and the number of years in which a shock would impact a country’s exports (1–5 years) (Supplementary Information). Hence we classified the types of shocks by severity–low (3 countries cease exports) or high (15 countries cease exports)–as well as by duration–short (1 year) or long (5 years). Additionally, in the case of multiple shocks, a gap of 2 years occurs between shocks^13, 21–27, 29, 49, 78^.

Previous analysis has most often considered the effect of a shock on a static network where nodes are removed, and an evaluation of the shock impact immediately afterward is carried out^15, 65, 75, 76, 79^. However, our focus was on the effect of shocks on the wheat trade network as it evolves in time, to ascertain the impact of shocks over a larger time frame. In some cases, the impact of shocks on trade networks has been measured by dynamically redistributing volumes of trade^1, 13, 38, 76^. As we have the simpler case of an unweighted network, no trade volume redistribution was considered. The effect of exogenous disturbances to our networks was ascertained by introducing shocks of different duration and severity during the process of network formation and evaluating their impact on network metrics.

Several metrics were used to determine network vulnerability and resilience, as well as the extent to which a shock has impacted a network. Average path length indicates the speed at which a shock will disseminate through a network, with a small average path length indicating quick spread, as most nodes in the network will tend to be near each other^3, 80^. The robustness to errors and vulnerability to attacks displayed by disassortative networks necessitates measurement of the assortativity coefficient^60–62^. The average clustering coefficient is a measure of cliquishness within networks, with clustering reducing network efficiency and resulting in increased vulnerability to attacks^61, 64, 66, 70^. The sizes of the GSC and GWC are respectively lower and upper bounds on the maximum size of a shock, given that the shock begins in the giant component^17^.

When networks have a positive symmetry metric, high symmetry, heterogeneity, and density contribute to network resilience^71^. Networks with power-law degree distributions are “scale-free,” and are robust where errors are concerned but are extremely fragile with regards to attacks^65, 66^. This is because their high degree heterogeneity means removing highly connected nodes results in rapid increases in network diameter and eventual network fragmentation^65^.

## Results

### Comparison of Empirical and Model Networks

**Figure 2:**
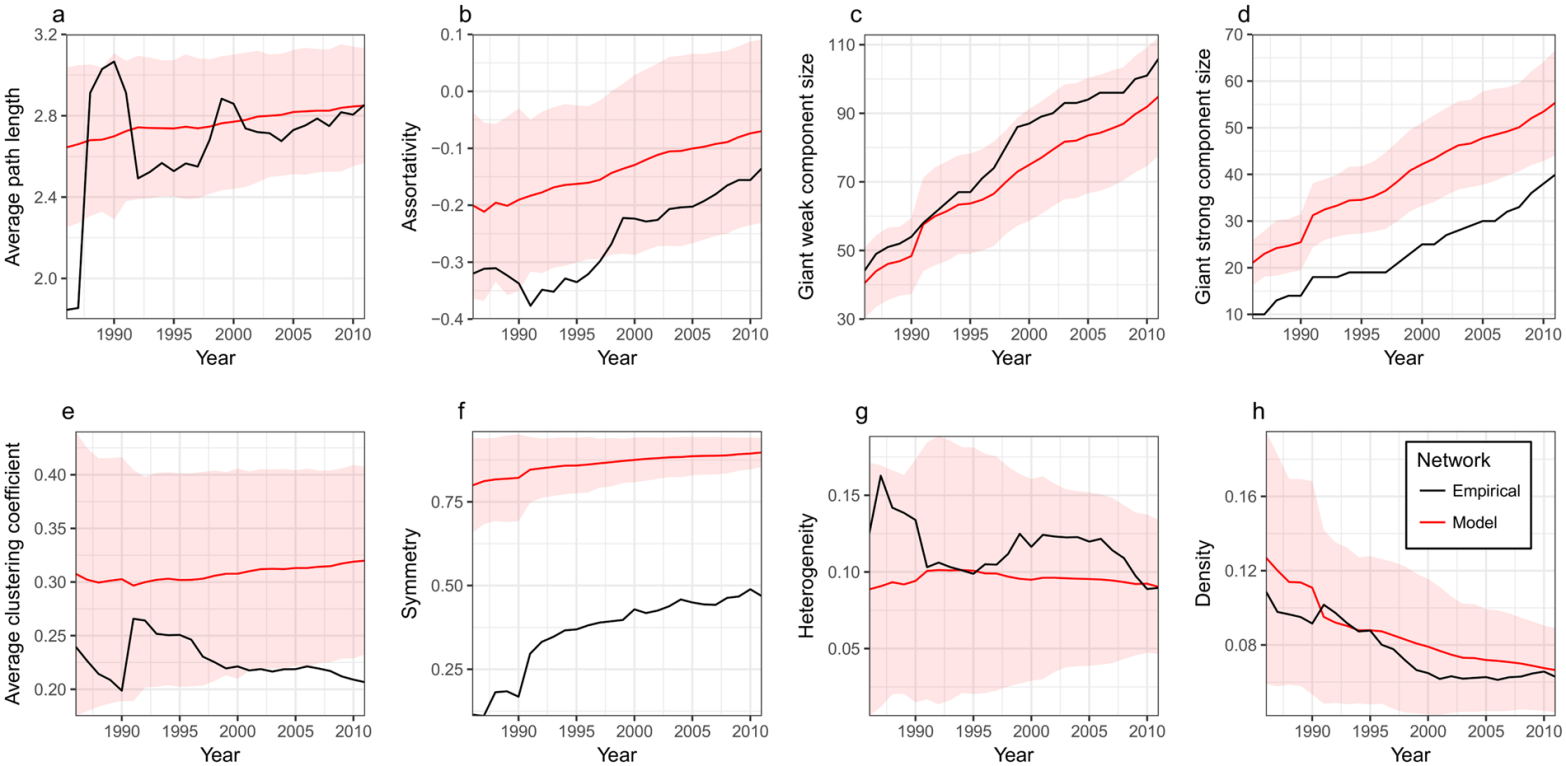
Selected empirical and model network metrics, 1986–2011. Red lines represent the mean metrics for model networks generated using the top 100 parameter sets ranked by least MSE normalized over assortativity, number of nodes, and reciprocity. The envelopes represents a range of ±2 standard deviations from the mean metrics for the model networks. Black lines represent the metrics of the empirical network.

Fitting a PA model to an empirical network evolving over several decades while using as few parameters as possible to avoid over-fitting the data is challenging. Nevertheless, the 100 best parameter sets provide a reasonable fit to the empirical network metrics (see Supplementary Fig. S5), as well as to the rest of the measured metrics (Fig. 2), from 1986–2011 both qualitatively and quantitatively. For the majority of metrics the empirical data falls within 2 standard deviations of the mean of the model networks. The GSC size and symmetry for model networks, while not displaying a good fit quantitatively, nevertheless display similar trends to those for the empirical network (Fig. 2c,g). From 1986–2011, the global wheat trade network experienced several shocks^13, 20–27, 29, 49^. Thus, discrepancies between metrics in the empirical and model networks may be impacted by the fact that our network simulations did not include shocks during calibration. Due to the influence of the assortativity coefficient on network vulnerabilities we also fitted the model using only assortativity as a measure of goodness-of-fit in an attempt to more closely match the metric in the empirical network. While this led to a better quantitative fit to the assortativity coefficient the increasing trend was lost (see Supplementary Fig. S10) so we proceeded with the best parameter sets ranked by the normalized MSE.

The addition of network rewiring steps that decay in number over time (Fig. 2) suggests that preferential attachment with an additional random component drives network formation. The fact that a decaying number of rewiring attempts leads to a good fit regarding network assortativity (Fig. 2b) suggests that as the size of the network increases, fitness plays a larger role in how edges are formed, as compared to random chance.

Calculation of the Kolmogorov-Smirnov statistic revealed that we should reject the hypothesis that the degree distributions for our empirical and model networks containing more than 100 nodes fit a power-law distribution (KS statistic calculated at the 5% significance level for each year in which number of nodes exceeded 100). As Konar *et al*. found that the degree distribution of the global VWTN was fit well by an exponential decay distribution with the decay coefficient given by the average degree of the network, we re-calculated the KS statistic to determine whether the same was true of our networks^33^. These calculations revealed that the exponential decay distribution provided a good fit to our network’s degree distributions at 5% significance, measured yearly. As a result, we cannot draw conclusions about network vulnerability using the scale-free property. However, because exponential networks also have a highly skewed node degree distribution, other network metrics can be utilised to make many of the same judgments, as we will subsequently show.

To illustrate the similarities between the model and empirical network, we visualized a single-year snapshot of the empirical continuous wheat trade network (Fig. 1a) and an exemplar model network (Fig. 1b) for 2013. These networks show several similarities including complex structure and heterogeneous degree distributions (Fig. 1c).

### Shocks and Model Network Growth

**Figure 3:**
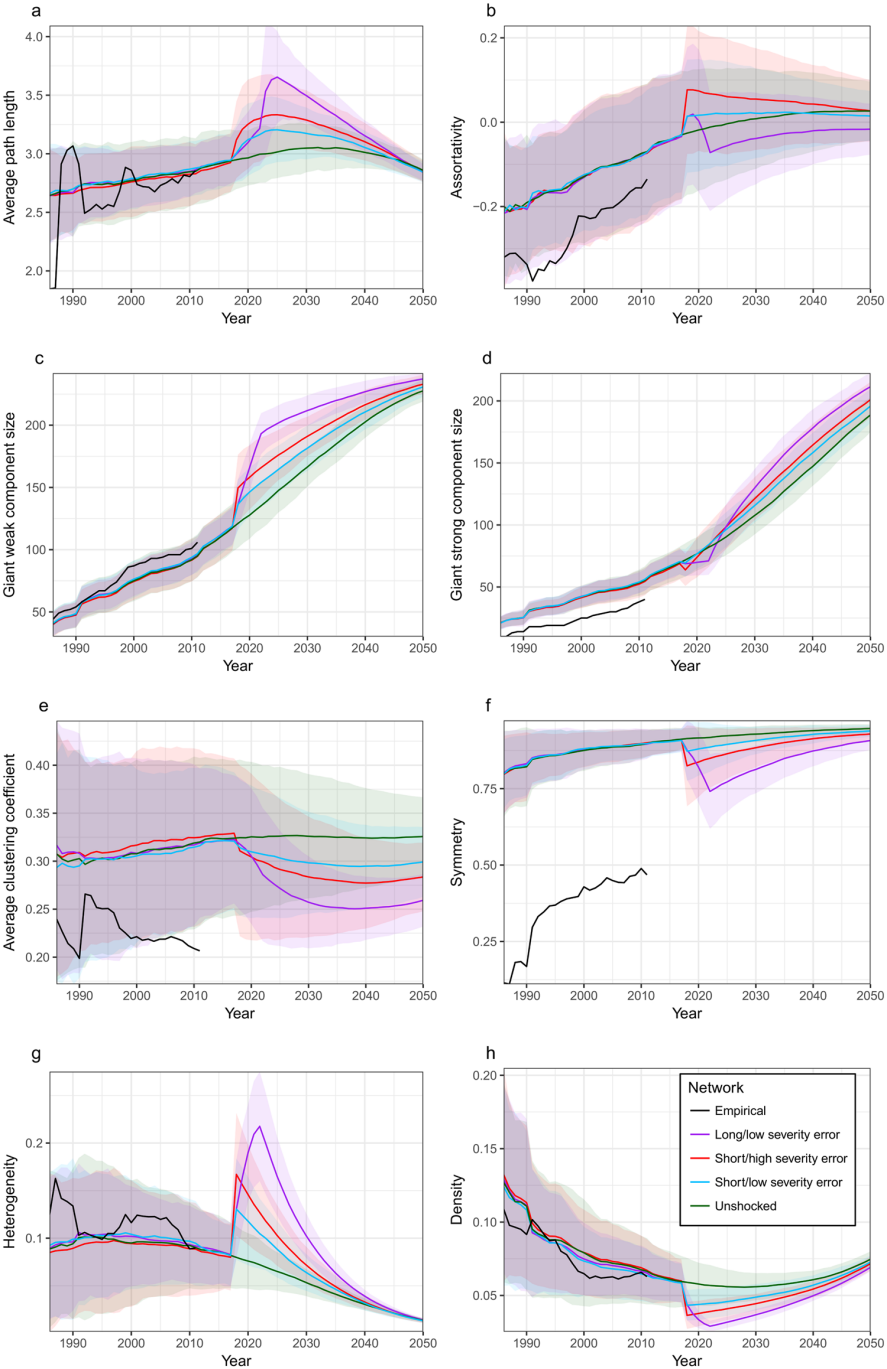
Impact of future errors on model network metrics. Shocks occurred in 2017–18. Coloured lines represent the mean metrics for model networks generated using the top 100 parameter sets ranked by least MSE normalized over assortativity, number of nodes, and reciprocity. The envelopes represent a range of ±2 standard deviations from the mean metrics for the model networks. Black lines represent the metrics of the empirical network using data from 1986 to 2011.

**Figure 4:**
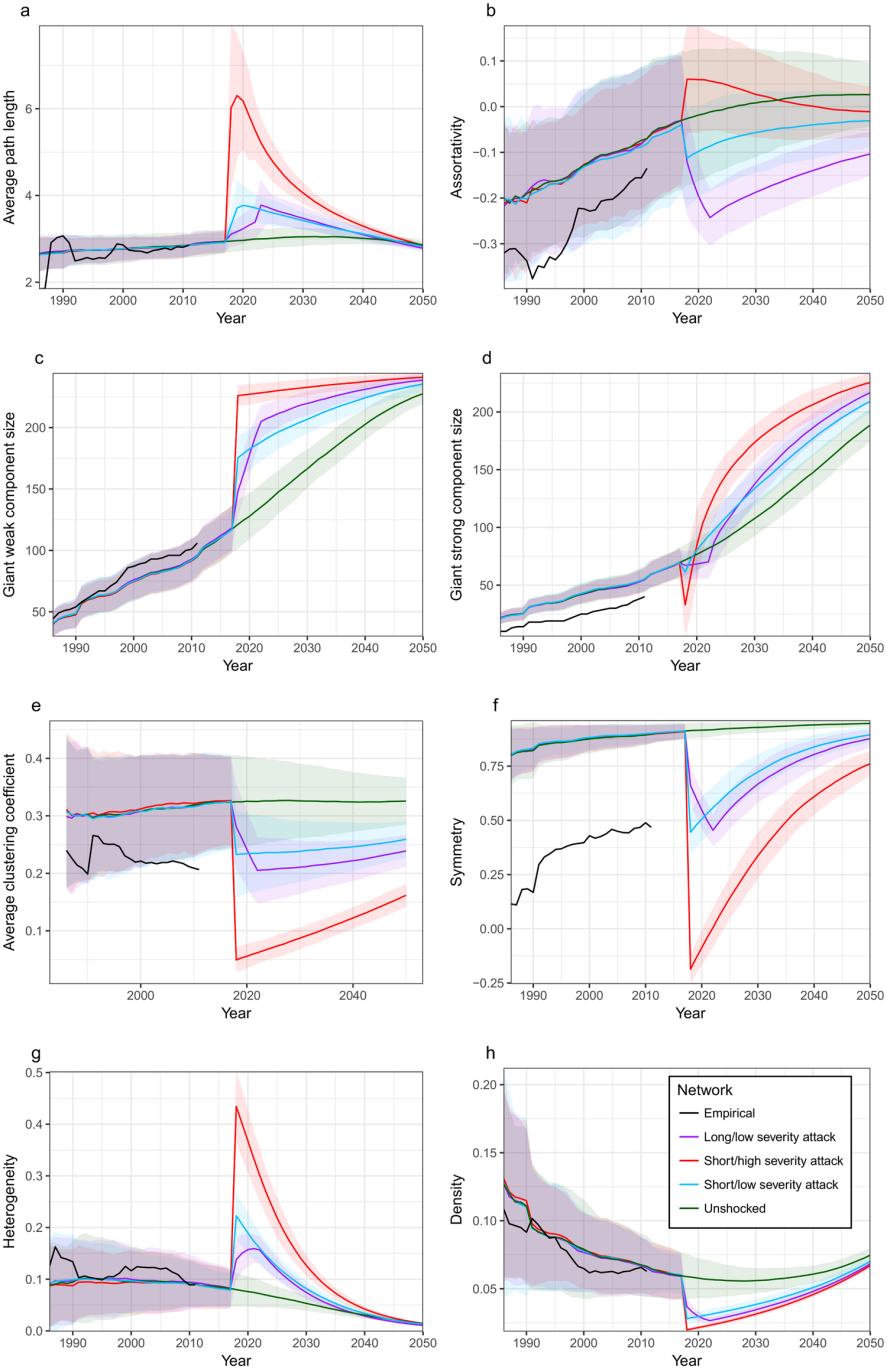
Impact of future attacks on model network metrics. Shocks occurred in 2017–18. Coloured lines represent the mean metrics for model networks generated using the top 100 parameter sets ranked by least MSE normalized over assortativity, number of nodes, and reciprocity. The envelopes represent a range of ±2 standard deviations from the mean metrics for the model networks. Targets with largest total degree are selected for attacks. Black lines represent the metrics of the empirical network using data from 1986 to 2011.

Notably, even shocks with low duration and severity that result in 1-year export bans for <2% of nodes have substantial and long-lasting effects on model network metrics (Fig. 3, Fig. 4). These impacts are especially heightened when networks experience attacks (Fig. 4). In the short term, networks affected by shocks will experience slower spread of subsequent shocks due to increased average path length (Figs 3a and 4a). They will also have a broader range of maximum shock sizes (higher maximum and lower minimum bounds due to changes in GWC and GSC size) (Figs 3c,d and 4c,d). Additionally, these networks will be more heterogeneous, and less dense and symmetric, resulting in short-term changes in network resilience (Figs 3f,g,h and 4f,g,h).

Increasing the severity or duration of a shock will increase its impact on network metrics. For errors, increasing the duration of a shock by 500% was more effective than increasing its severity by the same factor, with regards to changes in network metrics (Fig. 3). For attacks, the opposite was generally true: a severe shock had more influence on network metrics than a longer duration shock (Fig. 4). An exception to these trends is assortativity. While the error scenario leads to increases in assortativity for short duration shocks, long shocks result in an overall decrease this metric (Fig. 3b). Under an attack scenario, low-severity shocks cause a decrease in assortativity, while high severity shocks result in an increase (Fig. 4b). The long-term impacts of shocks on assortativity are much more durable for low-severity attacks than for high-severity attacks or errors of any severity and increased duration leads to a larger impact (Figs 3b and 4b).

The long-lasting effects of shocks on network resilience are minimal under an error scenario: by 2050 network heterogeneity, symmetry and density are approximately the same for shocked and unshocked networks (Fig. 3f–h). Similar trends in density and heterogeneity exist when a network is attacked (Fig. 4g,h). However, the long-term resilience of the network under attacks will be lowered due to the considerable and long-lasting decreases in symmetry (Fig. 4f). Shock type does not impact the upper bound on maximum shock size (by increasing the GWC size) in the long term, though attacks will result in a larger lower bound on shock size (larger GSC size) (Fig. 3c,d and 4c,d).

Interpreting the meaning of changes in assortativity and average clustering coefficients (ACC) lead us to a contradiction. Networks that have experienced attacks, and are thus more disassortative than networks that have experienced an error or are unshocked, should be the most vulnerable to any subsequent attack. However, based on ACC, attacks will result in networks that are the least vulnerable to subsequent attacks due to their low clustering (Figs 3b and 4b). It is unclear whether the lowered ACC or the increased disassortativity will have the dominant impact on vulnerability. This discrepancy is discussed further in the subsection: Consequences of Repeated Shocks.

As the network evolves it approaches a constant low level of assortativity and clustering, suggesting that the network is evolving to be less vulnerable to attacks (Figs 3b,e and 4b,e). As a result, the network’s resilience varies over time: symmetry increases and density and heterogeneity decrease, on the whole (Figs 3f–h and 4f–h). Low levels of network heterogeneity and density may indicate that poor network resilience will persist as we approach 2050 (Figs 3f–h and 4f–h). For both errors and attacks, networks show some long-term resilience (Figs 3a,g,h and 4a,g,h). Regardless of whether a shock has been introduced to the network, the majority of the 244 countries in the world are included in the GWC by the year 2050, though shocks increase the size of this component, as well as of the GSC (Figs 3c,d and 4c,d). As the giant strong component grows to include all countries in the world, a larger number of countries will have the potential to be impacted by shocks. Additionally, all shock types produce long-lasting reductions in network clustering, creating a less cliquish trade network (Figs 3e and 4e).

While the mean changes in network metrics are unique for each combination of shock type, duration, and severity, variability in the impact of a specific shock on the network means that a variety of different shocks can result in similar outcomes. This is especially clear for long-term predictions of the influence of errors: by 2050 there is a large overlap between the potential effects of all 3 error scenarios considered (Fig. 3).

### Consequences of Repeated Shocks

**Figure 5:**
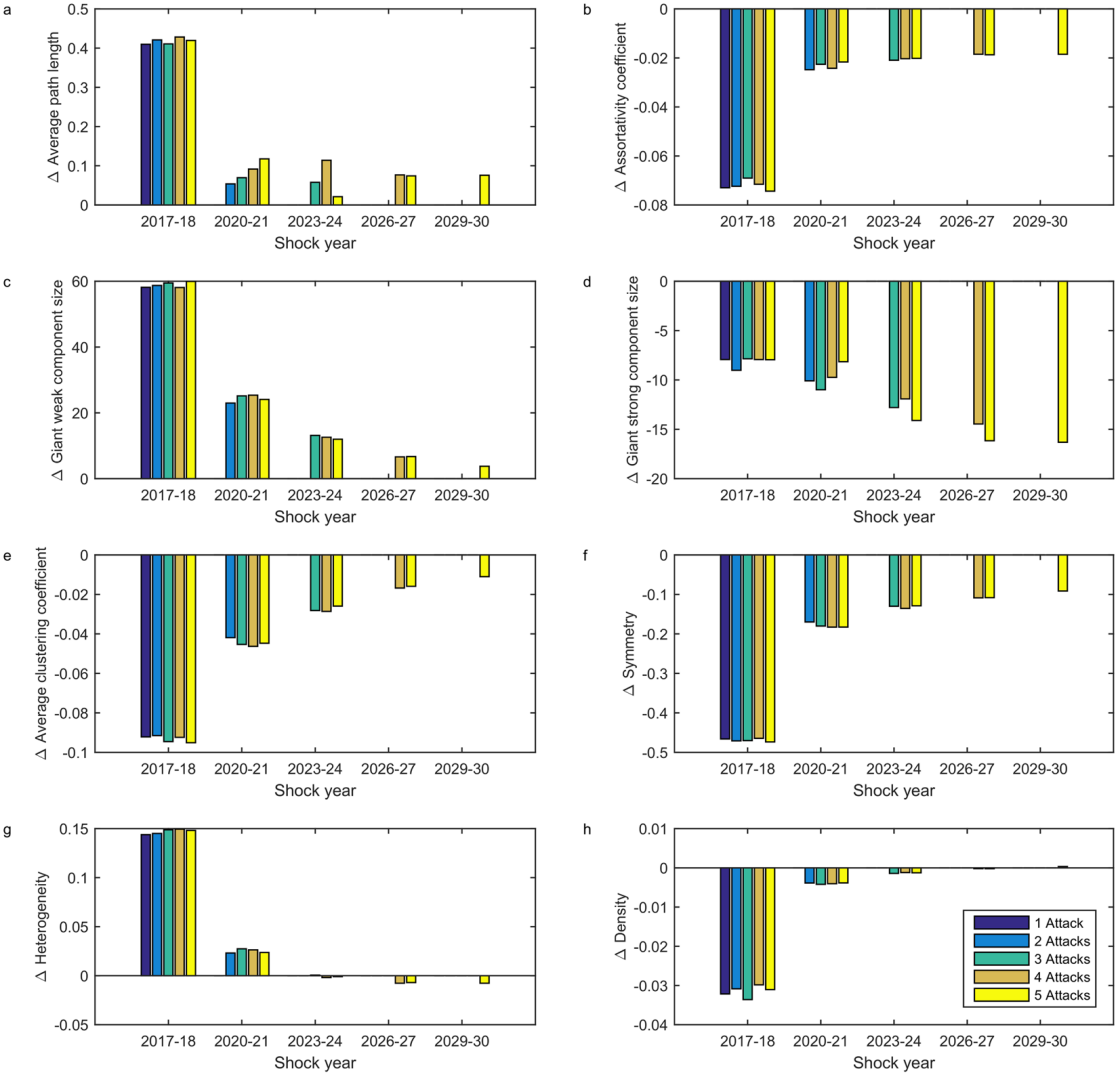
Impact of multiple attacks on model network metrics. All attacks are low severity/short duration, with a gap of 2 years between every attack. Bars represent the mean change in metrics for model networks generated using the top 100 parameter sets ranked by least MSE normalized over assortativity, number of nodes, and reciprocity. Bars are only shown for years where an attack occurred; changes caused by the temporal evolution of the network in unshocked years are not included.

We simulated the effect of repeated shocks, considering multiple shocks of the same type and sets of shocks in which both errors and attacks occurred. All shocks had the same duration. With respect to most of metrics, a previous attack reduces the short-term impact of subsequent attacks: each additional attack has a lesser impact on network metrics than the preceding attack (Fig. 5). This observation helps to resolve the contradiction described in the previous section–our results indicate that in the short term, for a network that has experienced an attack, decreased vulnerability to attacks resulting from lowered ACC outweighs increased vulnerability to attacks due to increased disassortativity. An exception to this trend is the giant strong component size which decreases by a larger amount with every subsequent attack (Fig. 5d).

Sequences of shocks that include the largest number of attacks will have the most substantial long-term effects on the network, leading to lasting changes across a broad range of metrics. For most metrics the type and occurrence of the first shock, as well as the type of the second shock, influence long-term outcomes (see Supplementary Fig. S11 and Supplementary Fig. S12). Average path length, heterogeneity, and density metrics show remarkable resilience. Regardless of the number, type, magnitude, and duration of shocks, by 2050, a shocked network be similar to an unshocked network in regards to these metrics (Figs 3a and 4a, see Supplementary Fig. S11 and Supplementary Fig. S12). As with the single shock scenarios, variation in the outcome of different types and combinations of shocks means that disparate shock scenarios may have comparable outcomes. For example, both double shocked networks that were initially attacked, regardless of the type of the second shock, show similar metrics as we approach 2050 (see Supplementary Fig. S12). Due to the short time between shocks (2 years), the natural growth process of the network does not significantly impact responses to shocks.

### Comparison of Attack Strategies

For attacks on our model networks the use of a sequential versus simultaneous attack does not lead to noticeably different outcomes on any time-scale in the duration/severity scenarios (see Supplementary Fig. S13). We also considered whether attacks that targeted the countries with the largest number of export links, instead of those with the largest number of trade links, would result in more damage to the network. However, for all duration and severity levels, targeting countries based on total degree and out-degree are equally damaging (Fig. 4, see Supplementary Fig. S14). This result suggests that countries highly ranked in terms of total degree will also be highly ranked by out-degree in our network, in general. This is true of the empirical network where there is a significant correlation between total degree and out-degree (see Supplementary Fig. S15).

## Discussion

We developed a dynamic model for the time evolution of the global agri-food wheat trade network to explore the impact of shocks on its structure and its response to subsequent shocks. In order to fit the model, we also constructed an empirical global wheat trade network consisting of continuous trade partnerships and analysed its network metrics. It was found that a preferential attachment model with network rewiring was able to replicate the temporal evolution of crucial metrics in the empirical network in many respects, despite not requiring country-level data such a GDP. Based on analysing time evolution of historical metrics in the empirical network and network model predictions of future trends, we predict that the network is evolving to be less vulnerable to attacks, though its resilience may remain low for the next few decades. We also experimented with applying shocks to the model network to determine the effect of factors such as extreme weather events or agro-terrorism on its dynamics. We found that even short-term shocks (1-year duration) have substantial and long-lasting effects on its structure, increasing the diversity of links as well as determining its response to subsequent shocks. While attacks have the largest impact on networks, for repeated attacks the damage from each subsequent shock will be smaller than from the last. Different network metrics are affected in different ways by attacks, but on the whole, attacks make the network less vulnerable to subsequent shocks.

Previous analyses of global trade networks have led to conflicting predictions regarding the food security risks associated with the globalisation of trade, and the potential for the disruption of trade. Global wheat and rice trade networks have been shown to be transitioning to a “robust yet fragile” state where they are vulnerable to attacks but robust against errors^13, 38^. Conversely, Sartori & Schiavo found that the world trade network’s time evolution, while resulting in increased connectivity, has not led to higher levels of instability^6^. Our predictions regarding the future of the continuous wheat trade network show a transition towards a more stable network configuration where countries maintain trade links with a diverse group of partners. This pattern of growth suggests that the network is evolving to be less vulnerable to attacks, in agreement with Puma *et al*., although attacks still have the potential to damage the network severely^13^. However, the network is vulnerable to errors, due to low levels of cliquishness and slight assortativity. Extreme climate events, such as floods, droughts, and heat stress, are predicted to increase in frequency, both globally and in regions of Europe where most of the world’s wheat is produced, over the remainder of the 21st century^50, 51^. Thus, strategies for lessening the impact of shocks on the network will become increasingly crucial to global food security and are an important area for future research.

The time evolution of the continuous wheat trade network’s resilience is less clear than the time evolution of vulnerability due to opposing trends in resilience-related metrics. Network density is predicted to decrease over the next decade but will begin to increase beyond this time period and contribute to higher levels of resilience. This increase in density will result from new links being added to the network much more frequently than new nodes. Despite high symmetry, low levels of network density and heterogeneity indicate that network resilience may be poor for a considerable portion of the next century. There is some debate as to whether the methods used to arrive at these metrics as universal measures of network resilience are as broadly applicable as has been asserted^81^. However, most of our conclusions do not depend on the interpretation of these metrics.

The network was predicted to respond differently to changes in duration and severity of shocks for attacks versus errors. This discrepancy may indicate that strategies for dealing with them should differ. For example, containing the spread of a shock should be a higher priority than facilitating recovery of impacted nodes when the network is affected by an attack, whereas in the case of an error, recovery should be the higher priority. However, the variability in the impact of a specific shock means that shocks of different type, severity, and duration may have similar impacts on network evolution. Attacks reduce network vulnerability to future shocks of the same type. While it would be preferable for the network to remain attack-free, any attack will create a positive externality by reducing the impact of a subsequent attack that occurs while the network is recovering. Longer gaps between shocks, allowing more time for network recovery, would reduce the overall impact of shocks. However, the frequency of shocks impacting agri-food trade networks is expected to increase during the 21st century, meaning that short gaps between shocks are a reasonable assumption^50, 51^.

The choice of sequential or simultaneous attacks did not significantly impact the effectiveness of an attack. However, this result is likely dependent on our network and the severity of attacks. Sequential attacks and attacks based on other centrality measures should be explored in future, as previous work has shown that these variations on the attack strategy can be substantially more damaging than simultaneous attacks, and those based on total degree centrality, for both simulated and empirical networks^64, 77^. As well, model extensions could provide further insight into mechanisms of network growth. For example, established exporters of a specific commodity are more attractive to potential importers, suggesting that preferential attachment based on out-degree should be considered^74, 82^.

While there appears to be a lack of analyses of the network effects of shocks on agri-food trade networks, analyses of shocks on financial networks are consistent with our simulations. Reductions in network density and symmetry following a shock are evident both in our model networks and in international financial networks following the 2008 financial crisis^83^. The global banking network also exhibited a reduction in density following the financial crisis, and showed significant changes in several network metrics up to 4 years after the crisis, suggesting protracted effects from shocks^84^. In addition to these global effects of shocks, the impact of export bans can have long-lasting effects for individual countries. For example, the 1997 outbreak of foot and mouth disease in Taiwan led to export bans and severely reduced the country’s pork export^85^. As of 2013, the average yearly export volume since 1997 was 0.02% of the average volume in the 8 years preceding the outbreak^48^. Additionally, Japan (one of the largest importers of Taiwanese pork pre-outbreak) was forced to seek alternative trade partnerships as a result of the shock. In 1996, Japan’s largest source of pork was Taiwan, but by 2005 they had ceased importing from Taiwan and had increased the volume of their imports from the United States, Denmark, and Canada. Japan showed similar shifts in trade partnerships in response to the outbreak of Avian Influenza in Southeast Asia in 2003–2004^85^. In addition to illustrating the long-term effects of shocks, these case studies provide support for our assumed mechanism of network restructuring following a shock.

As noted in the model description, the number of nodes in the network must grow over time and the network must be disassortative for a preferential attachment model to be appropriate for describing network evolution. Some of our model networks become slightly assortative as we approach 2050. This may suggest that PA will decrease in importance as a driver of network growth, and models used for more long-term predictions should consider different mechanisms of network growth. Our work has focused on the wheat trade network, meaning that the impact of a shock that originated in another commodity network and spread to the wheat network, or vice versa, cannot be evaluated. One extension of this work could be to consider a multi-network model where the impact of a such a shock could be quantified. Additionally, it is important to note that the continuous network we have modelled does not include countries that only infrequently engage in wheat trade. As a result, we are unable to quantify the impact of shocks on these countries. However, our predictions indicate that the number of nodes in our network is approaching the number of countries in the world, thus decreasing the number of countries excluded from our model.

Finally, we suggest that a useful continuation of this work would be extending the model to include edge weights using methods from previous research^43, 46, 86^. This would give us a more complete picture of wheat trade network dynamics as we move toward 2050. As well, it would allow for more realistic predictions of shock outcomes. Not all trade partnerships would have the same volume and thus shocks impacting higher volume partnerships would have a larger impact on global trade than those impacting low-volume trades^8^.

This work demonstrates that the multi-year time evolution of specific commodity networks and the shocks that afflict them can be mechanistically modelled to provide insight into dynamics and response to future shocks under various possible scenarios. Future research should continue developing these models for the trade networks of other major agri-food commodities so their dynamics can be compared and contrasted to gain greater insights into the dynamics of empirical networks.

## Electronic supplementary material

Supplementary Information

## Data Availability

The datasets used to inform our model fitting and analysis are available in the FAOSTAT repository, http://www.fao.org/faostat/en/#data/TM. The datasets we generated are available from the corresponding author on request.
