# Author Correction: Dynamics of the Global Wheat Trade Network and Resilience to Shocks

**DOI:** 10.1038/s41598-019-42458-6

**Published:** 2019-04-24

**Authors:** Kathyrn R. Fair, Chris T. Bauch, Madhur Anand

**Affiliations:** 1 University of Waterloo, Department of Applied Mathematics, Waterloo, N2L 3G1 Canada; 2 University of Guelph, School of Environmental Sciences, Guelph, N1G 2W1 Canada

Correction to: *Scientific Reports* 10.1038/s41598-017-07202-y, published online 03 August 2017

The original version of this Article omitted an affiliation for Kathryn R. Fair. The correct affiliations for Kathryn R. Fair are listed below:

University of Waterloo, Department of Applied Mathematics, Waterloo, N2L 3G1, Canada

University of Guelph, School of Environmental Sciences, Guelph, N1G 2W1, Canada

This has now been corrected in the HTML and PDF versions of this Article, and in the accompanying Supplementary Information.

