# Supplementary material for: Dynamics of the Global Wheat Trade Network and Resilience to Shocks
Source: Sci Rep. 2017 Aug 3;7:7177. doi: 10.1038/s41598-017-07202-y (PMC5543146; doi:10.1038/s41598-017-07202-y)
Supplement: Supplementary file 1 — Supplementary Information [file 41598_2017_7202_MOESM1_ESM.pdf]

Dynamics of the Global Wheat Trade Network and Resilience to Shocks

Kathyrn R Fair[1,2,*], Chris T Bauch[1], and Madhur Anand[2]

[1] University of Waterloo, Department of Applied Mathematics, Waterloo, N2L 3G1, Canada
[2] University of Guelph, School of Environmental Sciences, Guelph, N1G 2W1, Canada
[*] k3fair@uwaterloo.ca

Supplementary Information

Supplementary Methods

Defining a Continuous Trade Network

Previously, weighted representations of agri-food trade networks and the associated virtual water trade network have been simplified by considering a backbone network. This backbone includes only the dominant links that are responsible for the majority (generally 80%) of the total trade volume[1–5]. We have taken a different approach, considering only continuous trade partnerships to simplify the model.

We define a continuous trade partnership in the 1986-2013 data set as an import or export that has occurred in at least 3 consecutive years as of the end of 2013; i.e. one has occurred $\forall\, t \in [a, 2013]$ where $a \leq 2011$. A 3-year cut-off is employed to account for the fact that the number of nodes and edges in the network drastically increases if partnerships that existed in 2012 and 2013, or only in 2013 are included (Fig. S1a, b). To account for the fact that shocks to the empirical network may have interrupted the continuity of otherwise well-established trade relationships, all partnerships for which trades have occurred in 90% or more of the years since they were initiated are included. The 90% threshold ensures that an otherwise continuous long-term relationship, spanning decades, will not be excluded from the network due to a lack of trade in 1 or 2 years. Since the majority of shocks to the wheat network, and other staple crop networks such as rice and maize, only persist for a single year, this translates to roughly 1 or 2 shocks[6–13]. The continuity condition has the added benefit of ensuring that countries that have ceased to exist since 1986 are excluded from the network, a logical assumption if any predictions of the future of the network are to be made.

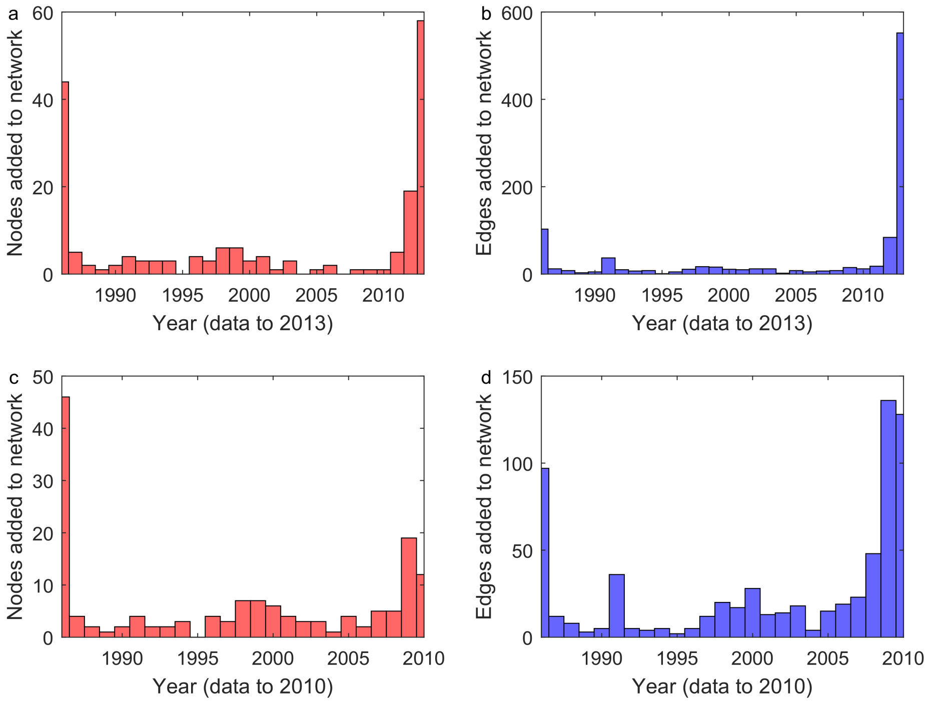

Figure S1. Number of nodes and edges added to empirical continuous wheat trade network, by year. (a) Year in which some country (node) i first initiated a continuous trade partnership with another country (data to 2013). (b) Year in which a continuous trade partnership (edge) between countries i and j was initiated (data to 2013). (c) Year in which some country (node) i first initiated a continuous trade partnership with another country (data to 2010). (d) Year in which a continuous trade partnership (edge) between countries i and j was initiated (data to 2010). Plots (a) and (b) use data for 1986-2013, (c) and (d) use data for 1986-2010. For all cases, the 90% threshold on continuity is applied, and an export partnership is considered as separate to an import partnership.

We explored the effect of a different final year of data on the growth of the continuous trade network. We see a similar trend if we exclude the final 3 years of data and create a network based only on 1986-2010 data (Fig. S1c, d). In both cases, a jump

in the frequency of entry for both nodes and edges occurs in the final 2 years, where the 1- and 2-year trade partnerships appear. This entry spike reconfirm our finding that many partnerships do not persist for more than 2 years. Including those which exist only from 2012 onwards would lead to the inclusion of many trades which fail to persist over longer time periods.

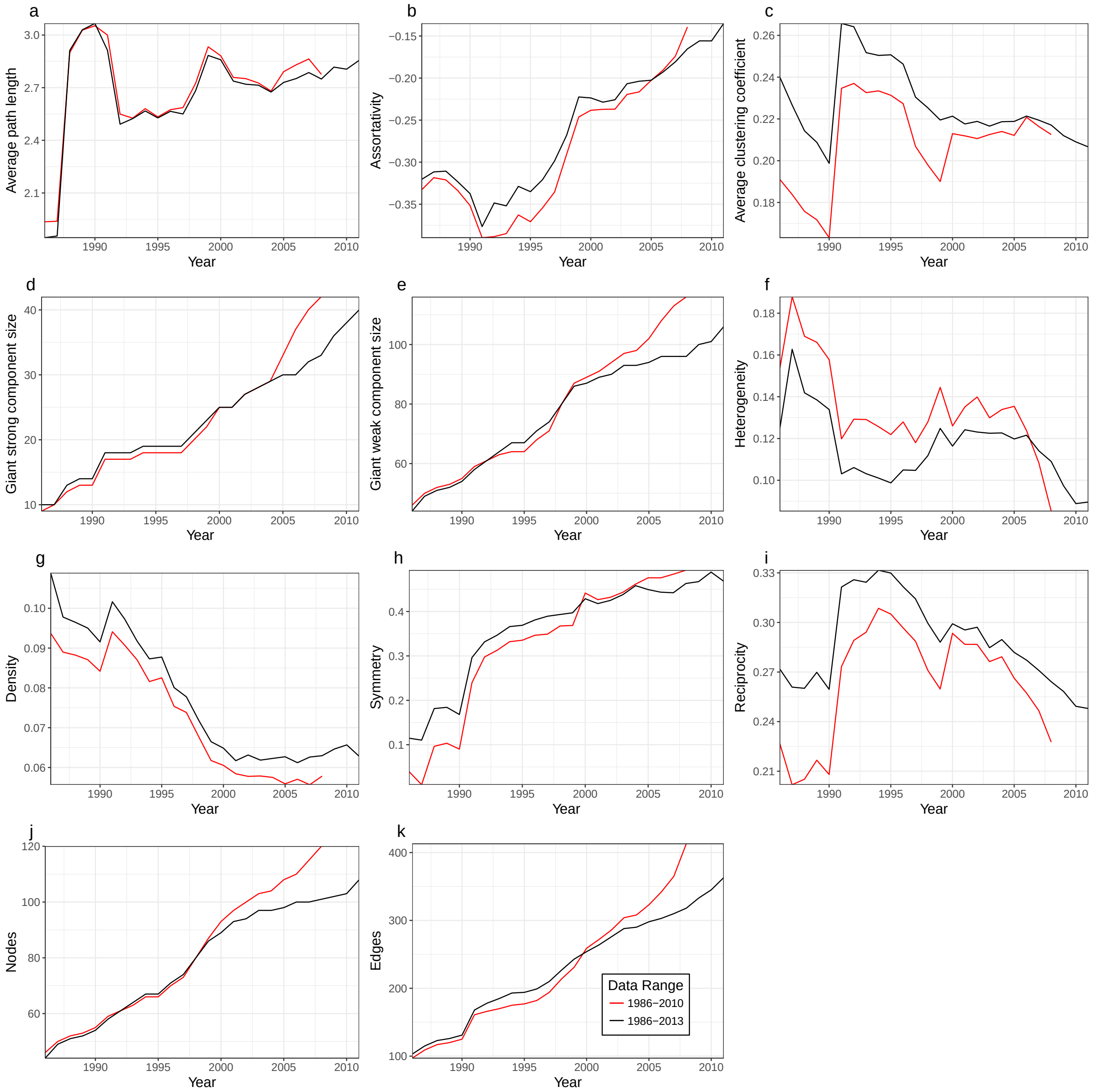

Figure S2. Comparison of metrics for empirical continuous wheat trade network using different final years. Both networks used a 3-year cut-off for continuous trades, and were generated using empirical data,

We also considered how a different final year would impact the structure of the continuous trade network (Fig. S2). With regards to our fitting metrics (assortativity, number of nodes, and reciprocity), the 2010 and 2013 final year networks produce similar trends and metrics (Fig. S2b, i, j). The most noticeable difference between these choices of final year for fitting metrics is that the 2010 network contains more nodes (and edges) (Fig. S2j, k). This is unsurprising as countries which ceased trading and trade partnerships which ceased to exist between 2010-2013 would be in this network, but not in the 2013 network. Thus, a model network fitted using the 2010 data would have similar reciprocity and assorativity trends and metrics to one fitted using 2013 data, but contain more nodes and edges. For most other metrics used in our analysis (Fig. S2a, c, f-i), the 2010 and 2013 networks are quite similar in terms of trends and metrics, suggesting that a model informed by 2010 data would provide a reasonable fit to the 2013 empirical network. Exceptions here are the giant weak and strong component sizes (Fig S2d, e), which due to their close relationship to the number of nodes in the network, would likely be larger in a model fitted to 2010

data. These observations suggest that the largest impact of using a different final year will be on metrics related to the number of countries trading, and number of trades (number of nodes and edges, giant weak and strong component sizes). This analysis demonstrates that using the most recent year for which data is available as the final year leads to the most accurate description of the continuous trade network. By including data for the years 2011-2013 in our analysis we can exclude partnerships which were discontinued in these years and should no longer be part of the network.

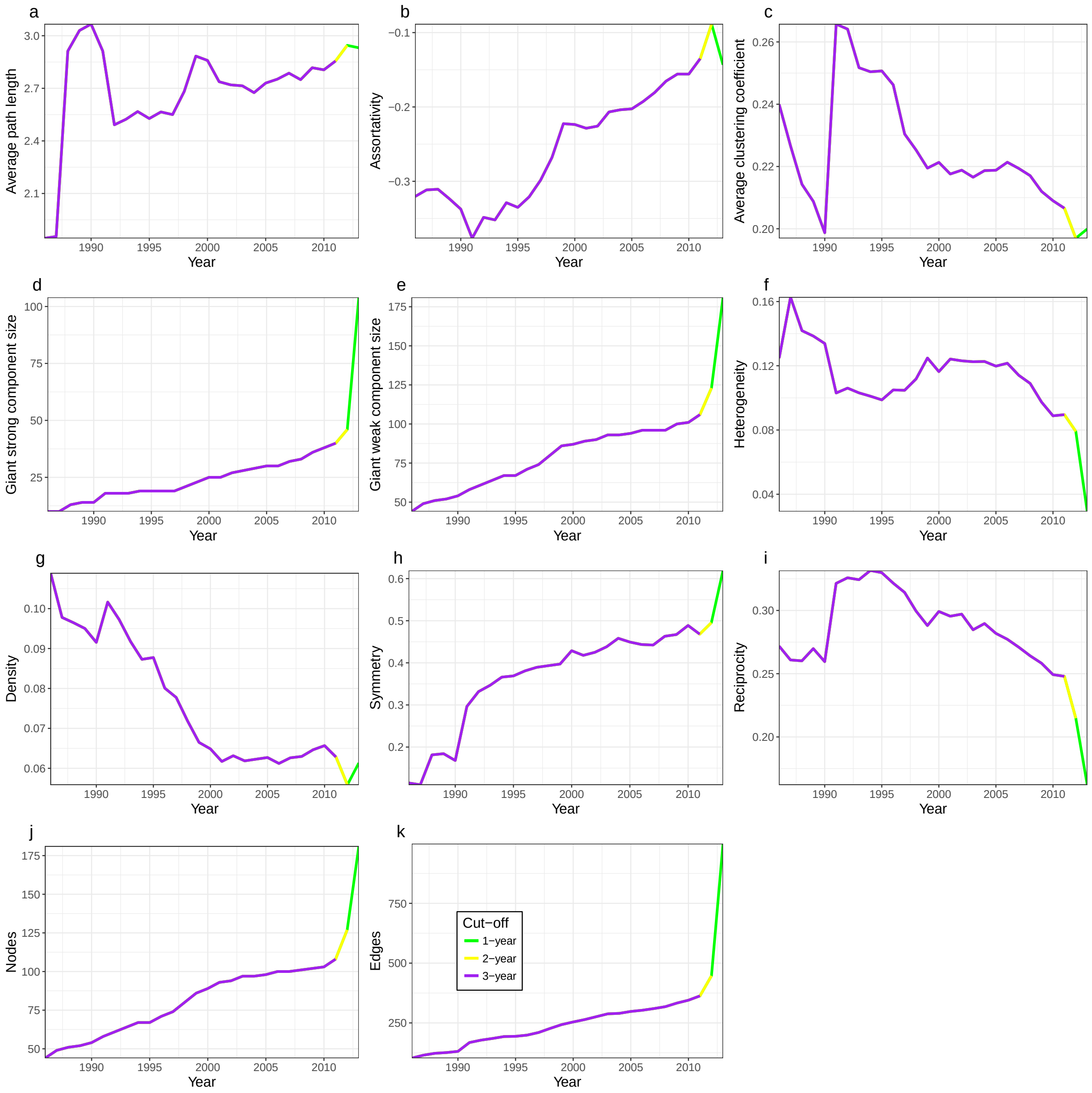

Figure S3. Comparison of metrics for empirical continuous wheat trade network using different cut-offs. Cut-offs correspond to the number of years a trade must have been established prior to 2013 to be considered continuous. A 1-year cut off can be interpreted as no restriction on length of partnership; here a trade is considered continuous as long as the trade has occurred in 90% or more of years since it was established. This means that trade partnerships which only exist in 2013 are considered continuous for the 1-year cut-off case. All networks use empirical data for 1986-2013.

Finally, we explored how the network's structure would be impacted by reducing the cut-off threshold for a trade to be considered continuous (Fig. S3). Our network utilised a 3-year cut off, meaning a trade must have occurred in at least the years 2011-2013 to be included in the network. Here we consider how network structure would differ if trade only needed to occur in 2012-2013 (2-year cut-off) or 2013 (1-year cut-off) to be included. It is clear from the substantial changes in most metrics that relaxing the cut-off impacts network structure. The alterations resulting from switching to a 2-year cut-off are noticeable, but a

further relaxation to a 1-year cut off has a drastic impact. Large increases in the number of nodes and edges in 2012 and 2013 shown in Fig. S1a,b are also evident here (Fig S3j, k). Other metrics are also affected; the size of the giant weak and strong components increases substantially (Fig. S3d, e), and there are drastic changes in heterogeneity, symmetry, and reciprocity (Fig. S3f, h, i). These changes result in large deviations from the trends for 1986-2011. This analysis suggests that while relaxing our cut-off to 2 years would have some impact on the structure of the network we are modelling, results would be similar to those we present for the parameter fitting using a network with a 3-year cut-off. However, if the cut-off was reduced to 1 year, it is probable that the group of parameter sets which provided the best model fit to the empirical network would be substantially different. However, relaxing our cut-off would increase the probability that we are including trade partnerships in our network which do not persist beyond 2013. This reduces the value of our model predictions; if shocks impact nodes which should not be included in the network, the resulting changes in network metrics may not accurately reflect impacts on the empirical network. Additionally, it is likely that obtaining a good model fit would become difficult due to the change in metric trends between 1986-2011 and 2012-2013. If we tighten the cut-off for continuous trade, requiring a partnership to last more than 3 years, we reduce the number of years of data available for the fitting process, which is likely to negatively impact the accuracy of the fit.

Estimating Rate of Network Growth

To estimate the passage of time during network formation, a logistic growth model was fit to the cumulative number of edges in the wheat trade network by year, $m(t)$, for 1986-2013[14]. This $m(t)$ cannot exceed the maximum possible number of edges in a directed graph with $n(t)$ nodes where $\max(m) = n(n-1)$[15]. We assume that the network can be fully saturated, or have total reciprocity, as the empirical network includes reciprocal trades (Fig. S4c). Using the 244 countries and dependent territories in the world as of 2015, $n_{max} = 244$, the largest possible network would contain $m_{max} = 59292$ edges[16]. This value was held constant throughout the fitting process, with the slope and midpoint of the sigmoid estimated using linear regression. A two-sample Anderson-Darling test (version 1) comparing the fit to data for 1986-2010 gives p=0.66728[17]. As $p > 0.05$, we concluded that both samples came from the same distribution at the 5% significance level.

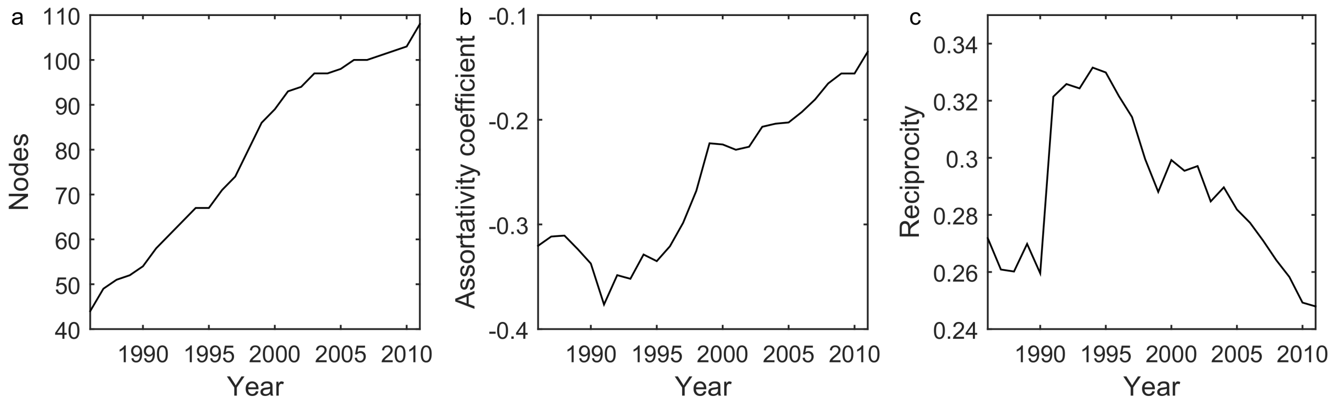

Figure S4. Time evolution of metrics for the empirical continuous wheat trade network (1986-2013). (a) Number of nodes. (b) Assortativity. (c) Reciprocity. Network generated using empirical data.

Using the fit we estimated the number of edges added to the network each year, running all simulations for $t \in [1875, 2060]$. The logistic growth model allowed us to introduce shocks with realistic durations. Even if no new countries enter the trade network post-2013, meaning $n_{max} = 108$, we would have $m_{max} = 11556$, a threshold not exceeded by our logistic model for $t \in [1875, 2060]$, though the parameters chosen for the logistic model with $m_{max} = 59292$ would provide a poor fit for the new upper bound on the possible number of edges. However, the number of nodes in the network has increased over time (Fig. S1a, Fig. S4a), and thus such a scenario is unlikely. We acknowledge that without empirical data on $m(t)$ for $t < 1986$ or $t > 2013$, it is difficult to justify the use of one functional form over another; it is possible that a linear or exponential function better describes $m(t)$ than the logistic form we assume.

Model Parametrisation

We ran approximately 5000 simulations for combinations of α, β, and ε from equation (1) as well as C and λ from equation (3), across ranges for all parameters. Ranges for α and β were set to $[1, 200]$, based on the parameters used in previous similar models[18,19]. For C, λ, and ε we experimentally determined ranges that led to reasonable model outcomes in terms of the network metrics. These ranges are $C \in [25, 1500]$, $\lambda \in [0.01, 0.1]$ and $\varepsilon \in [9.0 \times 10^{-5}, 1.2 \times 10^{-5}]$. There are no physical limits on the values these parameters may take; the limits we introduced facilitate a good fit to the metrics for the empirical

network. Once suitable ranges had been determined, we began testing parameter sets using large increments for all parameter ranges. This allowed us to focus on a smaller portion of the parameter space; $\alpha,\ \beta \in [1,5]$, $C \in [25,1025]$, $\lambda \in [0.01,0.05]$ and $\varepsilon \in [9.5\times10^{-5}, 1.15\times10^{-5}]$, where model outputs for assortativity, reciprocity, and number of nodes were reasonably close to those for the empirical network. Our 100 best networks had parameter sets drawn from these reduced ranges (Fig. S5).

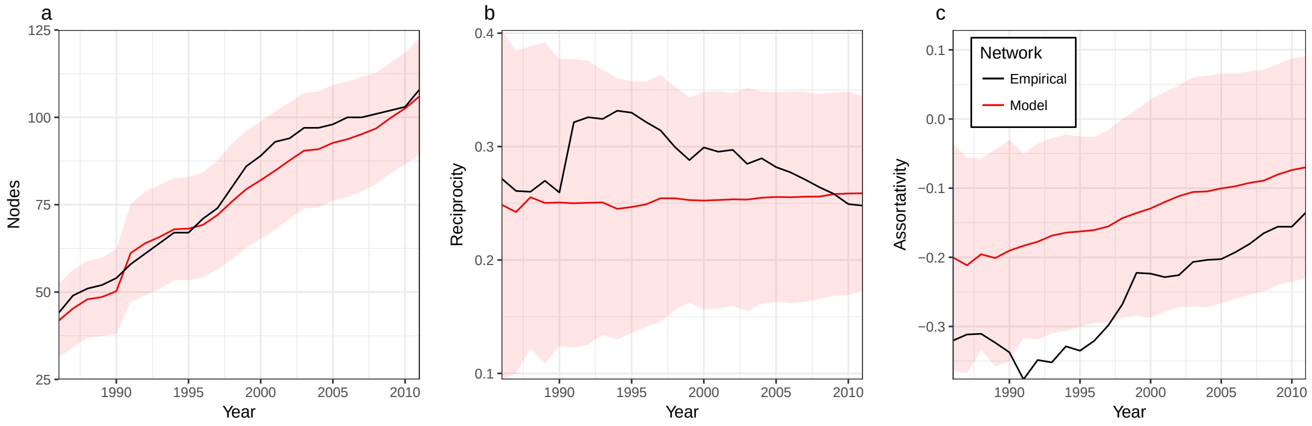

Figure S5. Network metrics for model parametrisation 1986-2011. Red lines represent the mean metrics for networks generated using the top 100 parameter sets ranked by least MSE normalized over assortativity, number of nodes, and reciprocity. The envelope represents a range of ±2 standard deviations from the mean metrics for the model networks. The black lines correspond to the metrics for the empirical network.

Implementing Shocks

For attacks, countries are targeted based on their connectivity. Connectivity can be determined using various centrality measures such as degree, out-degree, closeness, and betweenness centrality[20–25]. We define connectivity in terms of total degree centrality. This means that an attack will cause the sequential removal of the outgoing edges belonging to the countries with the highest total number of trade links. We limit ourselves to considering sudden shocks where production level adjustments occur on too long a timescale to counter their effects[26]. For the duration of a shock affected countries will not export; existing export links are removed, and new export links cannot be formed. After the shock, the affected countries, which no longer have any exports, can begin to establish new export links. Following the removal of export links for affected countries new links are formed to replace those removed. The formation of new links represents 2 occurrences whereby countries who previously imported from the affected countries seek to replace their lost trade volume; they may form new partnerships themselves or rely more on existing partnerships. This increased dependence on existing partners, in turn, means those countries either rely more heavily on *their* partners or form new trade links. While we do not explicitly model how the weights of trade flows are redistributed due to a shock, this rewiring of the network to account for changes in the availability of exports is conceptually similar.

Determining Realistic Shock Durations and Sizes

Each shock will be introduced for 1-5 years and impact 1-15 countries, as, depending on the shock, the duration and severity of it's impact will vary. For example, the late Victorian Great Drought of 1876-1878 had a protracted effect on global trade networks of rice products[4]. Another case, the 2001 outbreak of foot and mouth disease in the United Kingdom caused shocks to the global beef, sheep, and pig meat trade networks lasting approximately a year until the lifting of export bans in 2002[27]. Our ranges were drawn from a survey of the literature on recent export restrictions. These export restrictions include bans, quotas, and taxes resulting from food shortages that have influenced global trade. During the 2007/08 global food crisis, 15 countries (including 6 of the top 17 wheat exporters) restricted wheat exports, in some cases implementing complete export bans[4,6–10]. Additionally, rice export was restricted in 14 countries (including 4 of the top 9 rice exporters), grain export in 15, maize in 3, palm oil in 2, and oilseed & vegetable oils in 1[4,6–9,11]. Six of the top 17 wheat exporters (accounting for 90% of total trade) imposed some degree of trade restrictions, while 4 out of the top 9 rice exporters did so[4,28]. India maintained bans and restrictions on rice and wheat exports from February 2007 to September 2011[29]. In 2010/11 Russia, Ukraine, and Kazakhstan all implemented export restrictions on grain[12,13]. While not all of these restrictions have resulted in the complete cessation of exports, they provide a rough estimate of the appropriate size and length of shocks. Additionally, we use the 2007/08 and 2010/11 shocks as a template for our multiple shock scenario; for all cases of multiple, shocks a gap of 2 years will be observed between shocks.

Network Analysis For Agri-Food Commodities

Materials and Methods

Trade data for 549 commodities from 1986-2010 was obtained from the United Nations Food and Agriculture Organization's FAOSTAT database[14]. These commodities included wheat, as well as other crops, animal products, and crop-derived products. A $N \times N$ trade matrix, where N is the number of countries in the network, was used to represent the trade network for each commodity. Trades were extracted from the FAOSTAT database of imports, within the 25 year period. All cases where a value of 0 was reported for a trade were excluded, as these are caused by rounding small trade values to 0, or due to missing data. We believe that these excluded trades would not significantly alter the overall commodity-specific networks, in regards to the metrics we measured. The $(i, j)^{th}$ element of the matrix represents the export of the commodity under consideration from country j to country i. The value of this $(ij)^{th}$ element is the volume of the export from country j to country i. When comparisons were carried out, commodity-specific networks were given equal weighting, regardless of their size, to remove any bias towards the larger networks. These matrices were used to calculate diameter, average path length (AvPL), assortativity, density, average clustering coefficient (transitivity), average degree (AvDeg), the unweighted normalized average betweenness centrality coefficient (BCC), and reciprocity. Finally, the number of nodes and edges and the total value of the trade conducted with each network were recorded. These values were averaged over all commodity networks.

We sought to determine what subset of the array of network metrics we calculated would be sufficient to describe an individual commodity network, to reduce the computational time required. To this end, we looked for correlations between groups of metrics; if several metrics are highly correlated, it is possible to measure a single metric from this group and use that as a predictor of the correlated metrics. To determine relationships between metrics, the Pearson product-moment correlation coefficient, r, was calculated for all possible pairs of metrics. The associated p-values used were adjusted using the Holm-Bonferroni method to reduce the frequency of "false positives" resulting from multiple comparisons. Using a similar method as Jamakovic *et al.* the number of metrics required to describe the network was reduced[30]. Any correlation with a p-value > 0.05 was discarded as not significant at the 5% level and those where $|r| \leq 0.6$ were discarded as weak. Once groups of highly correlated metrics were identified, 1 metric from each group was selected for further analysis, to avoid redundancy. All metrics not significantly correlated with others were kept. For the purposes of describing our results, Jamakovic *et al.*'s definitions of correlation strength were used: $0 \leq |r| \leq 0.3$ (no correlation); $0.3 \leq |r| \leq 0.6$ (mild correlation); $0.6 \leq |r| \leq 0.9$ (significant correlation); $0.9 \leq |r| \leq 1.0$ (strong correlation)[30].

Results

When network metrics were calculated to give correlations (Fig. S6) the number of commodity networks within the sample was reduced to 524. This reduction was necessary as some networks contained an extremely low number of nodes and edges, meaning certain metrics could not be properly calculated. Two groups of metrics had significant to strong intra-group correlation: a size group containing the average path length, network diameter, and betweenness centrality coefficient, and a connectivity group of average node degree, density, average clustering coefficient, reciprocity and the number of nodes and edges (Fig. S6). The highly correlated metric groups for the agri-food trade network are not the same as those found by Jamakovic *et al.* in their study of a variety of real-world networks, though there is some overlap[30].

It would be preferable to consider the relationship between metrics for multiple networks of trade for the same commodity (e.g. wheat), over the same time period, to reduce the number of metrics needed to describe that commodity's network at that time. However, in the absence of multiple realisations of the wheat trade network over the same period, it becomes necessary to utilise networks for multiple commodities. Additionally, the metrics chosen from this analysis; average path length, assortativity, and average clustering coefficient, are well-established metrics for describing networks[31,32].

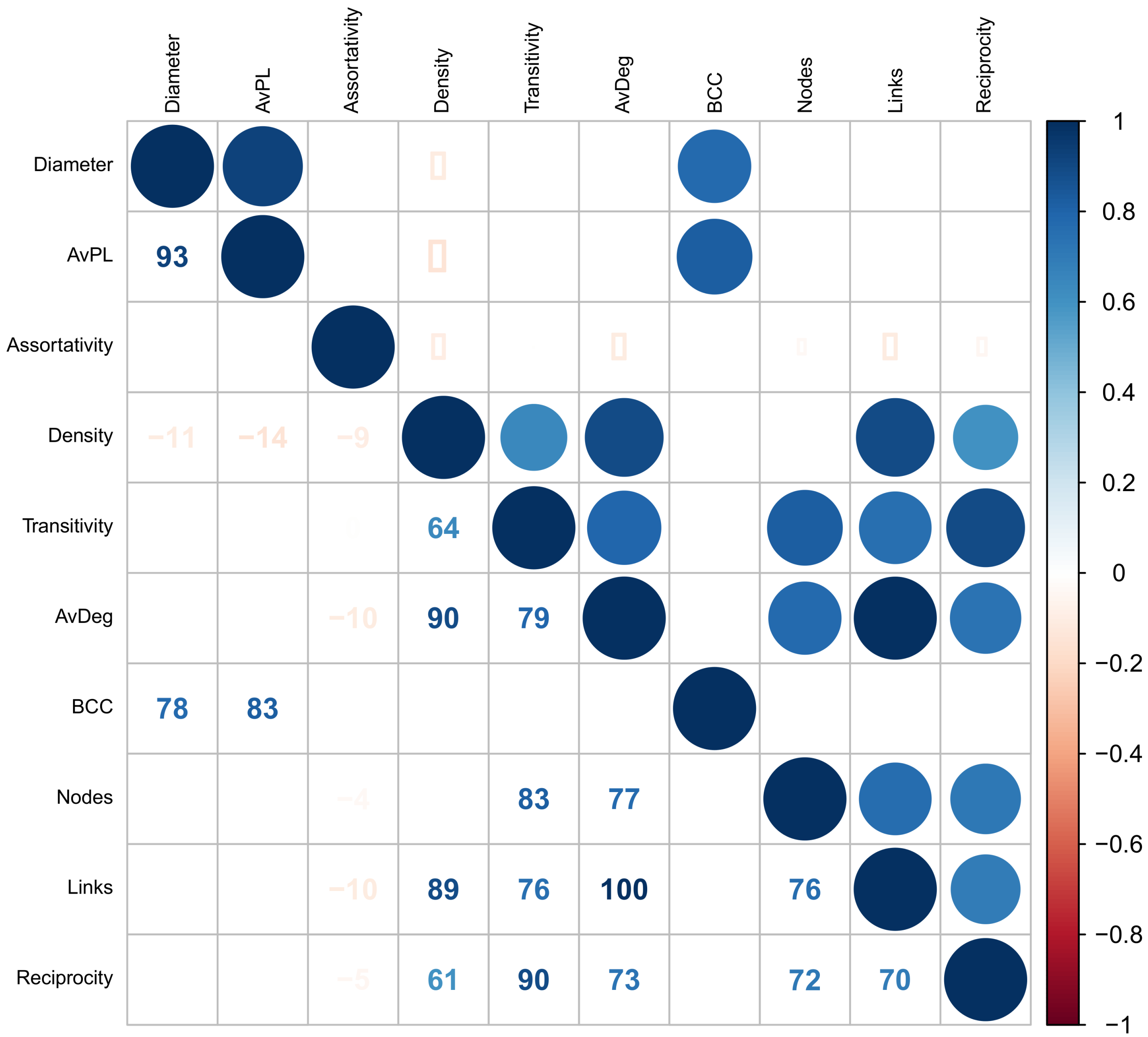

Figure S6. Correlation heat map for network metrics using 524 commodity networks for 1986-2010. All correlations shown are significant at the 5% level, non-Holm's-adjusted, and are given as % values.

Supplementary Figures

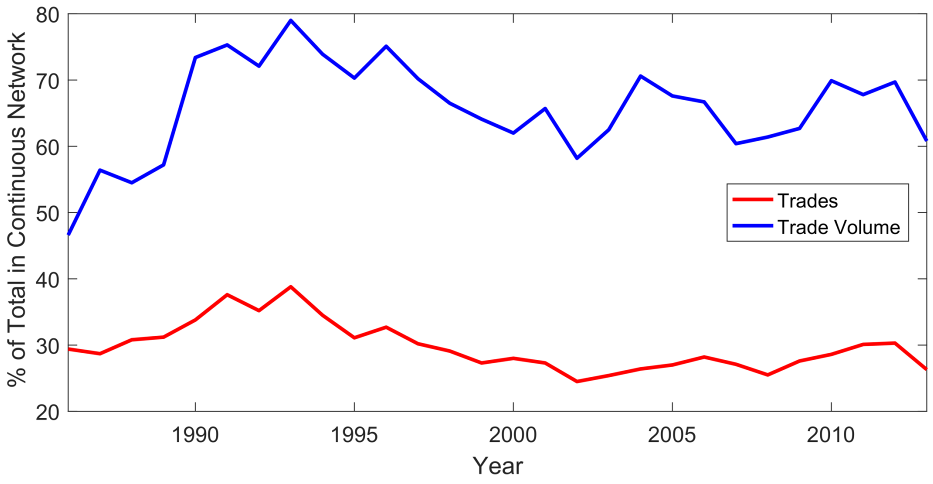

Figure S7. Comparison of empirical networks for total trade and continuous wheat trade by year. The red and blue lines represent, respectively, the percent of total trades which are included in the continuous trade network, and the percent of the total trade volume which is included in the continuous trade network, by year. Both networks are generated using empirical data.

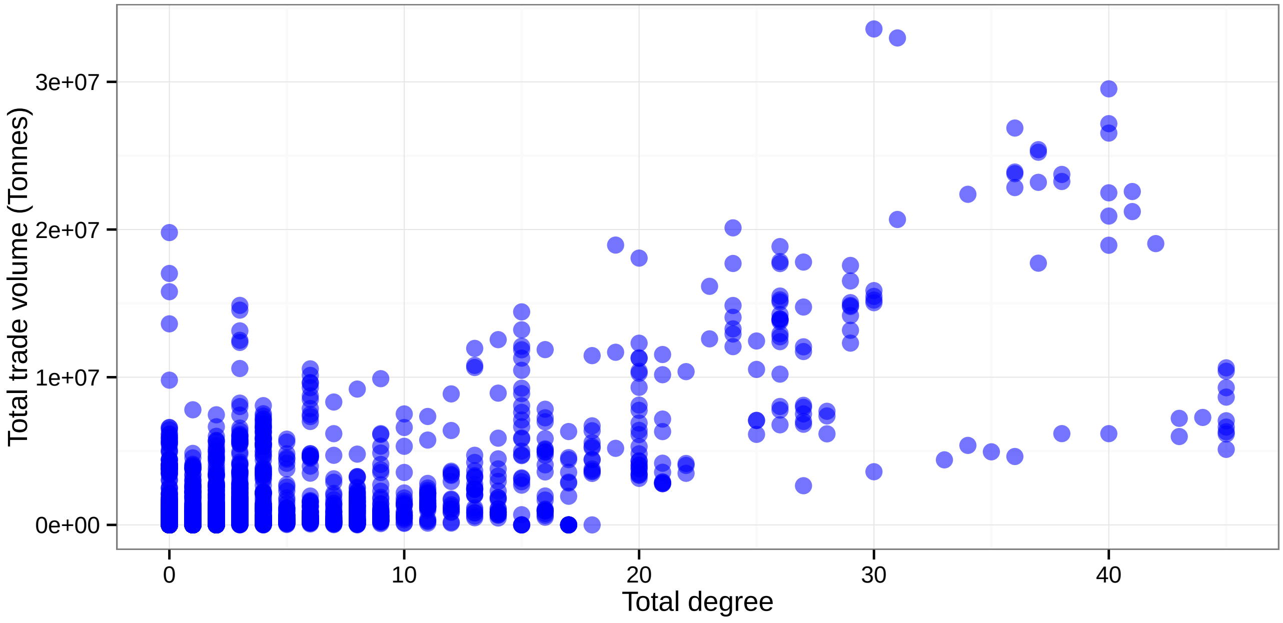

Figure S8. Relationship between total degree in the continuous wheat trade network and total trade volume from 1986-2011. Each point represents a single country in a single year, with a total degree of 0 corresponding to a country not included in the continuous trade network in that year, taken from empirical data. The darker the point, the higher the number of countries with that combination of total degree and trade volume. The correlation between total degree and trade volume is significant at the 5% level (p-value $< 2.2 \times 10^{-16}$).

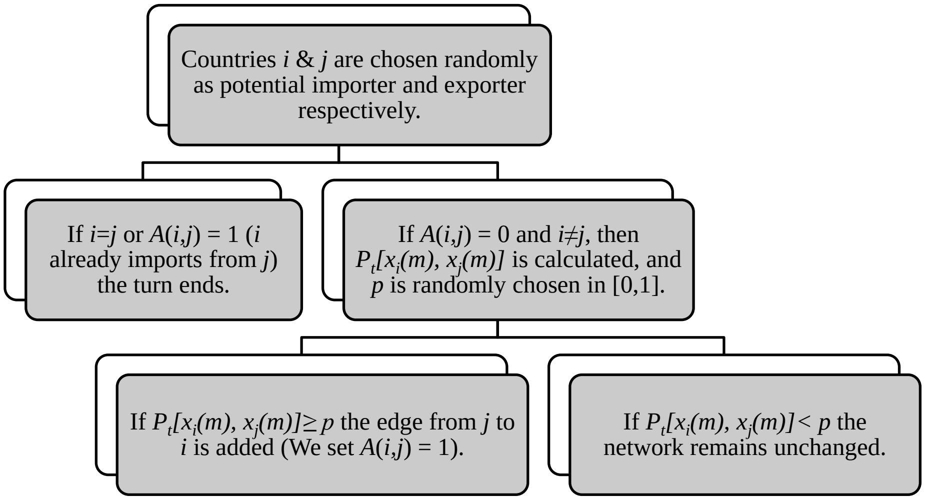

Figure S9. Decision-making process at each turn. If the final $P_t[x_i(m), x_j(m)] \geq p$ condition is not met, the turn ends with no new edges formed.

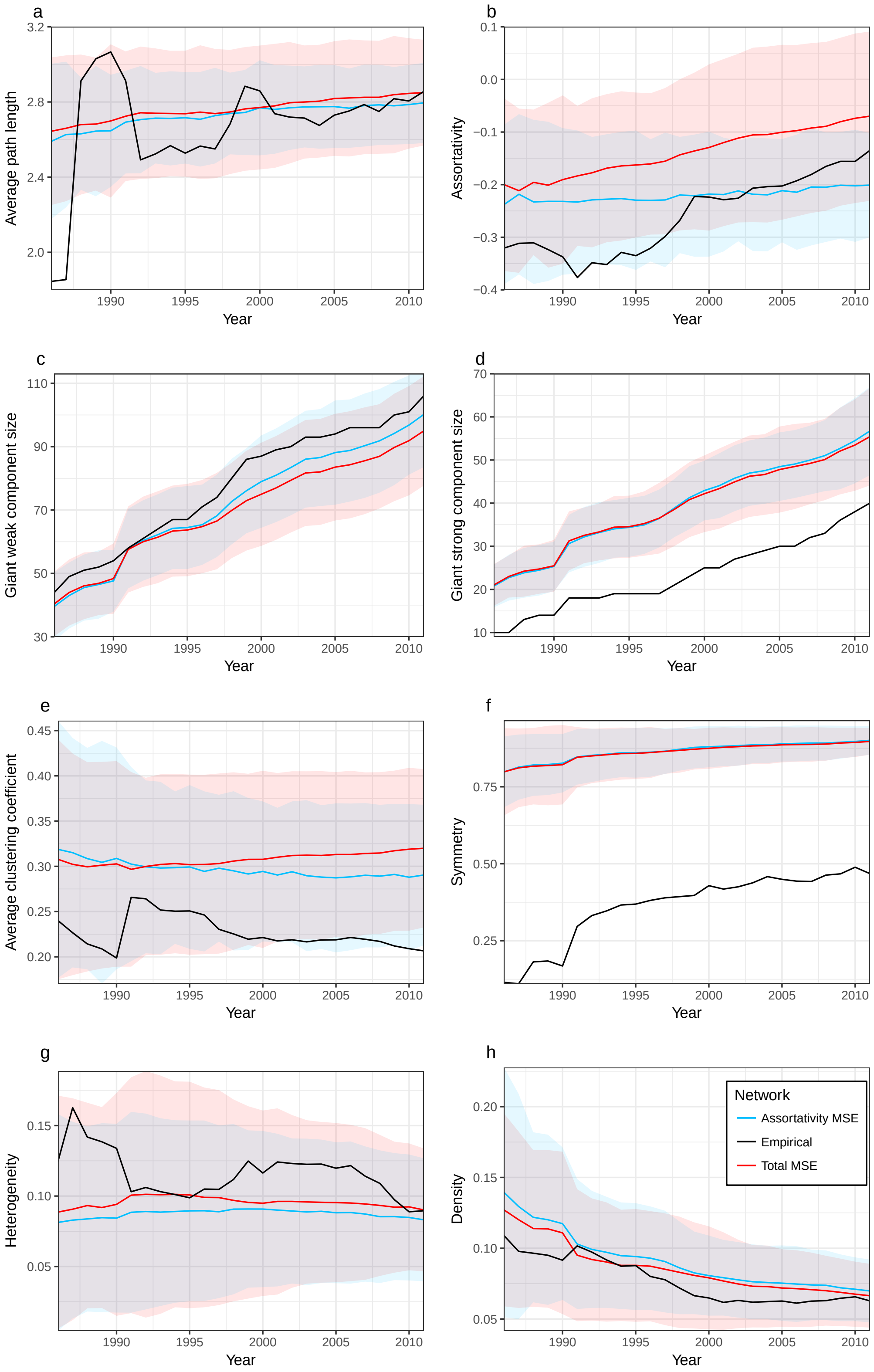

Figure S10. Selected network metrics 1986-2011. Coloured lines represents the mean metrics for model networks generated using the top 100 parameter sets ranked by least MSE for assortativity (blue) and normalized across assortativity, number of nodes, and reciprocity (red). The envelopes represent a range of ±2 standard deviations from the mean metrics for model networks. Black lines correspond to the metrics for the empirical network.

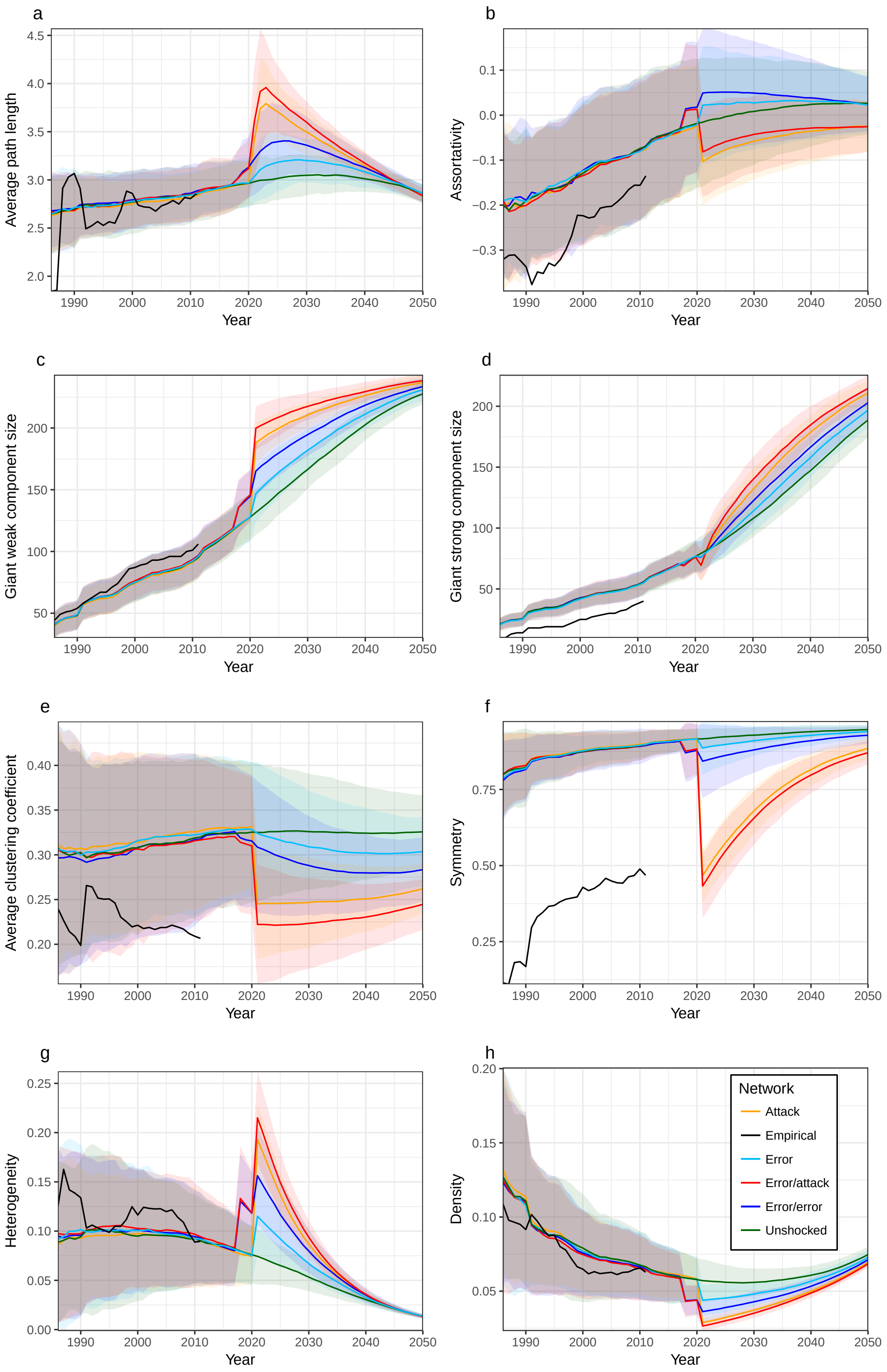

Figure S11. Impact of previous errors on changes in network metrics due to a subsequent shock. Initial error to double shocked networks (shown in blue and red) occurred in 2017-18, all networks shocked in 2020-21. All shocks were low severity/short duration. Solid lines represent the mean metrics for model networks generated using the top 100 parameter sets ranked by least MSE normalized over assortativity, number of nodes, and reciprocity. The envelopes represent a range of ± 2 standard deviations from the mean metrics for model networks. Black lines correspond to the empirical network.

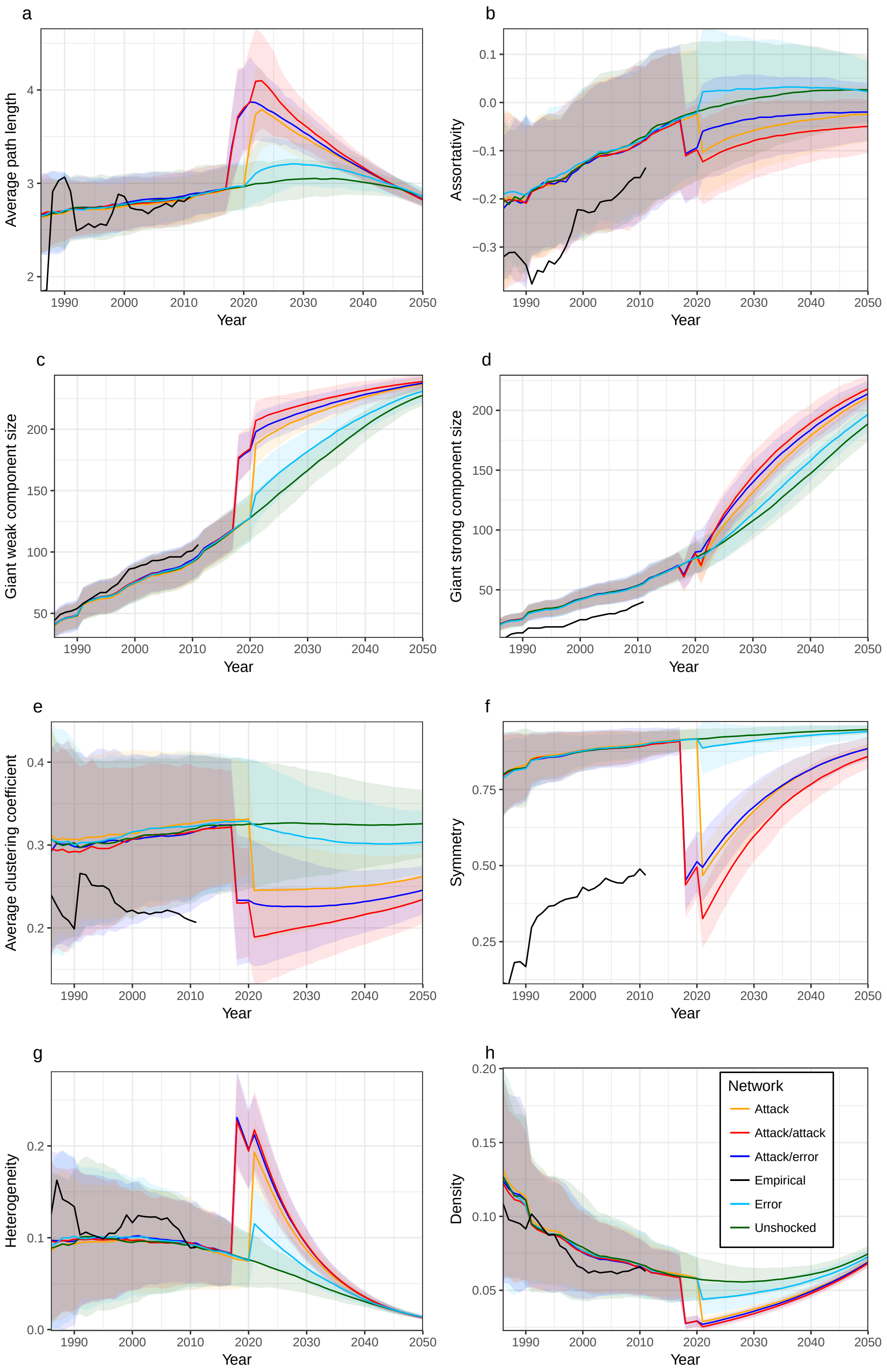

Figure S12. Impact of previous attacks on changes in network metrics due to a subsequent shock. Initial attack on double shocked networks (shown in blue and red) occurred in 2017-18, all networks shocked in 2020-21. All shocks were low severity/short duration. Coloured lines represent the mean metrics for model networks generated using the top 100 parameter sets ranked by least MSE normalized over assortativity, number of nodes, and reciprocity. The envelopes represent a range of ±2 standard deviations from the mean metrics for model networks. Black lines correspond to the empirical network.

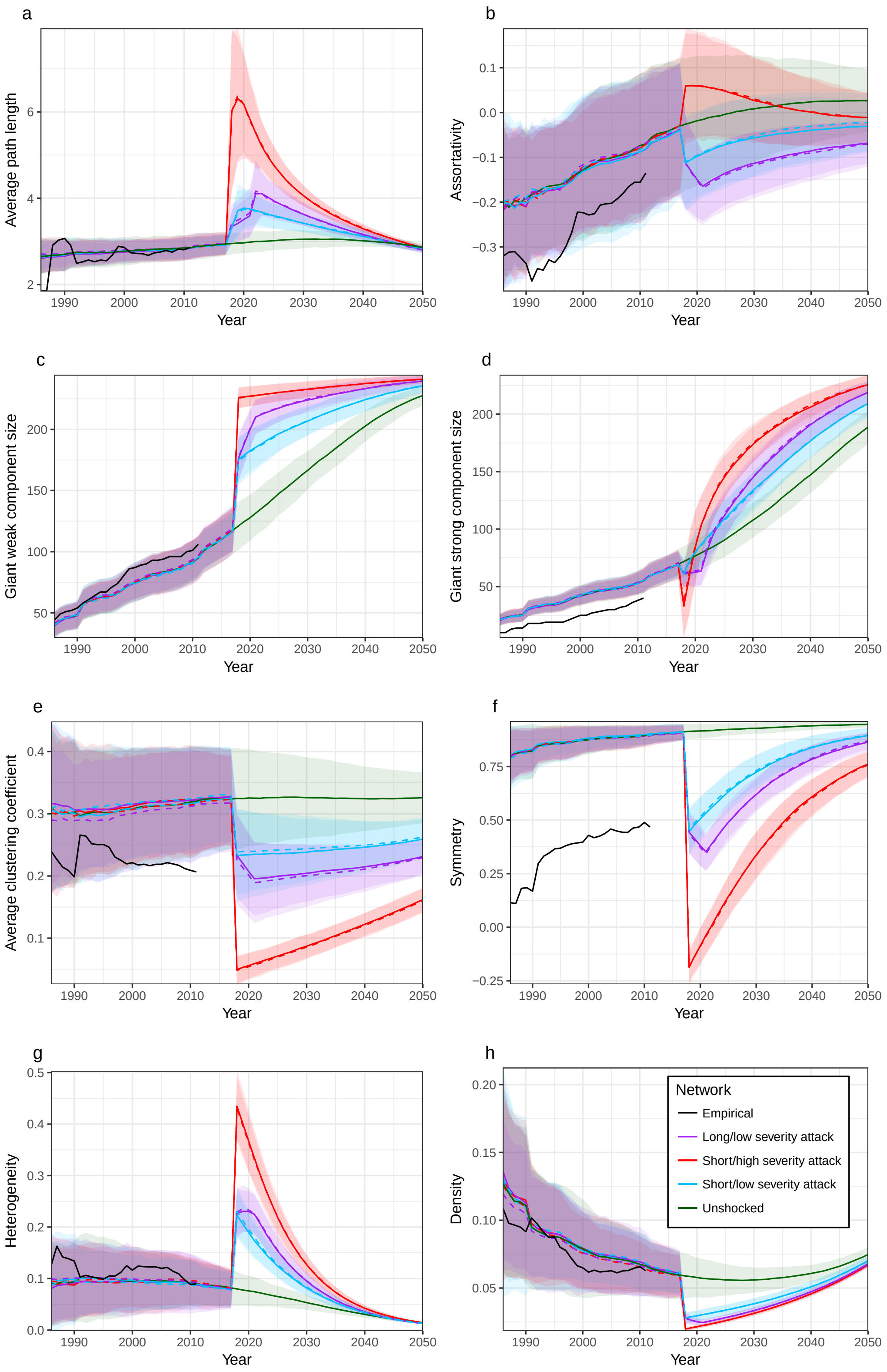

Figure S13. Comparison of sequential and simultaneous attacks. Shocks occurred in 2017-18. Coloured line represent the mean metrics for model networks generated using the top 100 parameter sets ranked by least MSE normalized over assortativity, number of nodes, and reciprocity. Dashed lines indicate a sequential attack, solid lines indicate a simultaneous attack. The envelopes represent a range of ± 2 standard deviations from the mean for model networks. Targets are selected by largest total degree. Black lines correspond to the empirical network, all other networks are generated using the model.

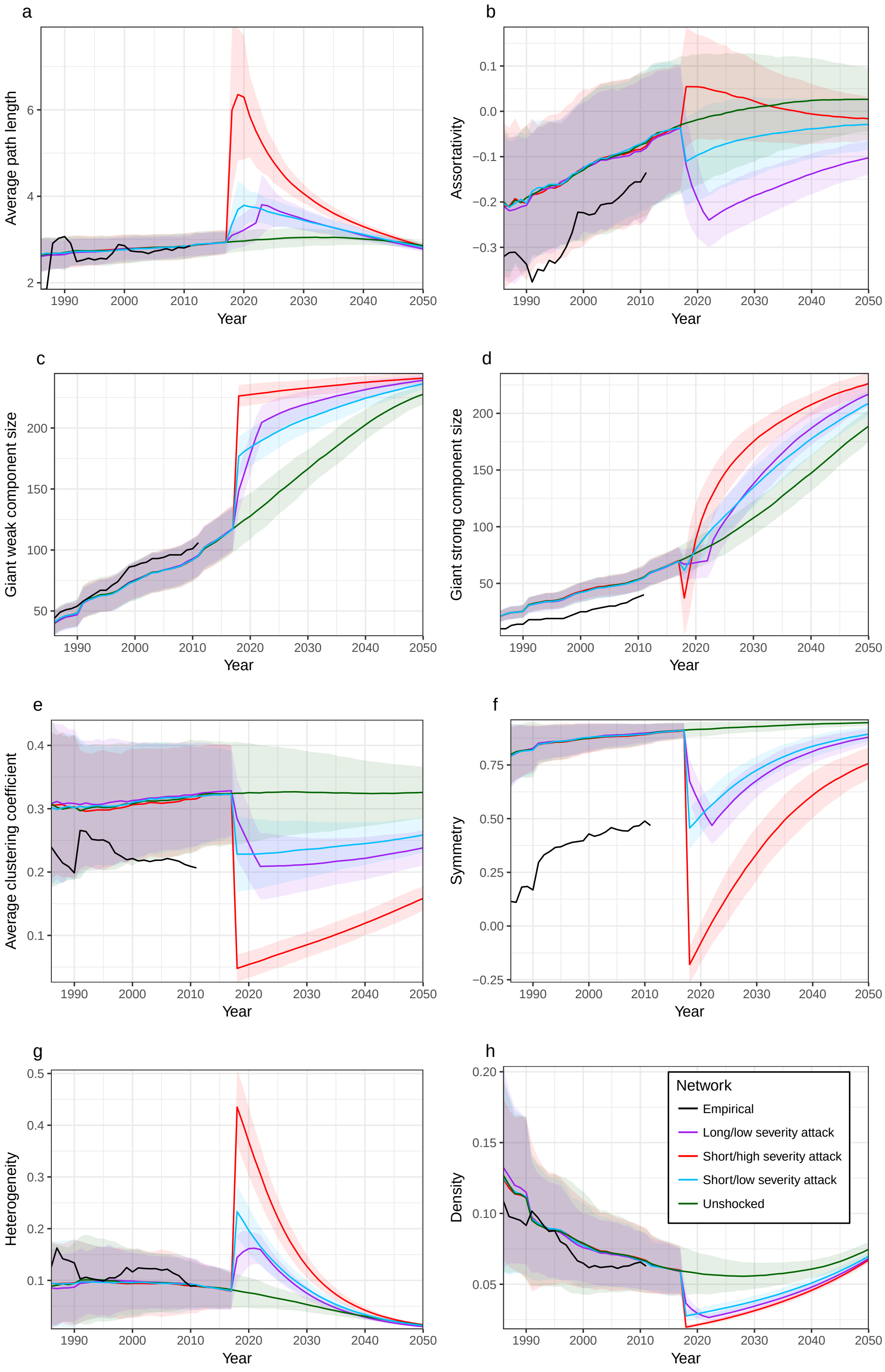

Figure S14. Impact of out-degree attacks on network metrics. Shocks occurred in 2017-18. Coloured lines represent the mean metrics for model networks generated using the top 100 parameter sets ranked by least MSE normalized over assortativity, number of nodes, and reciprocity. The envelopes represent a range of ± 2 standard deviations from the mean metrics for model networks. Targets are selected by largest out-degree. Black lines correspond to the empirical network.

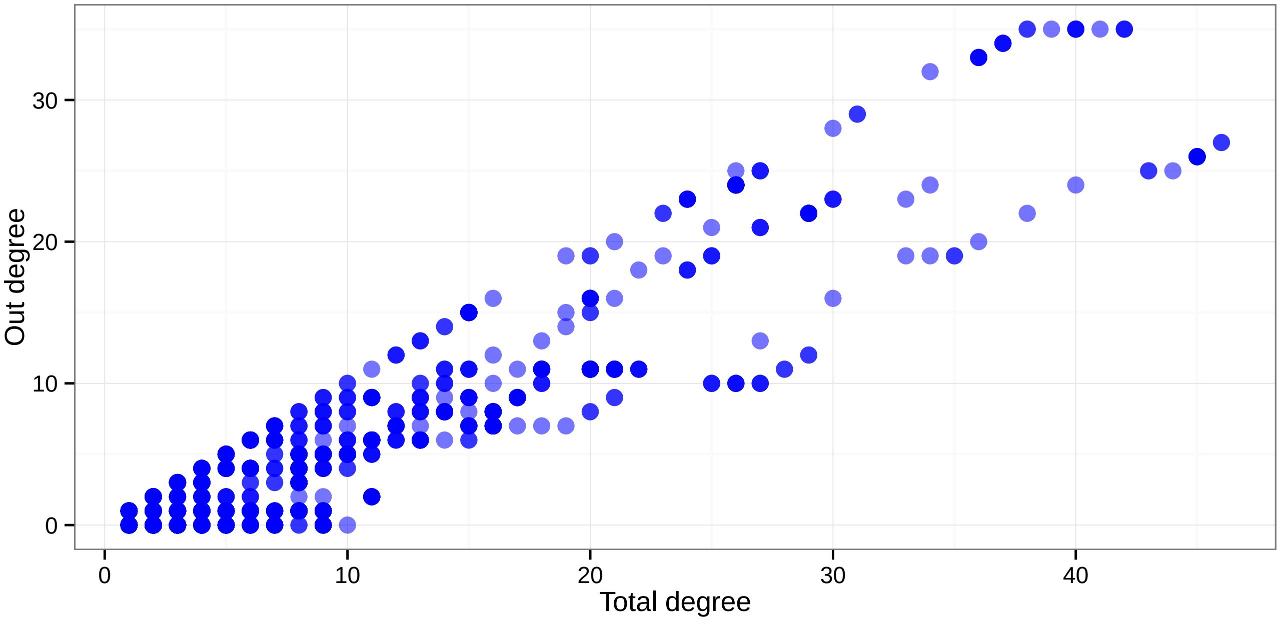

Figure S15. **Relationship between total degree and out-degree in the continuous wheat trade network from 1986-2011.** Each point represents a single country in a single year, taken from empirical data, with a total degree of 0 corresponding to a country not included in the continuous trade network in that year. The darker the point, the higher the number of countries with that combination of total and out-degree. The correlation between total and out-degree is significant at the 5% level (p-value $< 2.2 \times 10^{-16}$).

References

1. Ercsey-Ravasz, M., Toroczkai, Z., Lakner, Z. & Baranyi, J. Complexity of the international agro-food trade network and its impact on food safety. *PLoS ONE* **7**, e37810; 10.1371/journal.pone.0037810 (2012).
2. Konar, M. *et al.* Water for food: The global virtual water trade network. *Wat. Resour. Res.* **47**, 5; 10.1029/2010WR010307 (2011).
3. Glattfelder, J. B. & Battiston, S. Backbone of complex networks of corporations: The flow of control. *Phys. Rev. E* **80**, 36104 (2009).
4. Puma, M. J., Bose, S., Chon, S. Y. & Cook, B. I. Assessing the evolving fragility of the global food system. *Enviro. Res. Lett.* **10**, 024007 (2015).
5. Suweis, S. *et al.* Structure and controls of the global virtual water trade network. *Geophys. Res. Lett.* **38**, 10; 10.1029/2011GL046837 (2011).
6. Mitra, S. & Josling, T. Food export restrictions: review of the 2007-2010 experience and considerations for disciplining restrictive measures. *IPC Position Paper Agricultural and Rural Development Policy Series* http://www.ictsd.org/downloads/2009/02/exportrestrictions_final.pdf (2009).
7. Trostle, R. Global agricultural supply and demand: factors contributing to the recent increase in food commodity prices. *USDA Economic Research Service* http://www1.eere.energy.gov/bioenergy/pdfs/global_agricultural_supply_and_demand.pdf (2008).
8. Demeke, M., Pangrazio, G. & Maetz, M. Country responses to the food security crisis: nature and preliminary implications of the policies pursued. *FAO Economic and Social Development Department* http://www.fao.org/documents/card/en/c/506db788-bb64-4715-a211-1448442ed637/ (2009).
9. Timmer, C. P. Causes of high food prices. *ADB Economics Working Paper Series* https://www.adb.org/sites/default/files/publication/28375/economics-wp128.pdf (2009).
10. Dollive, K. The impact of export restraints on rising grain prices. *U.S. International Trade Comission Office of Economics Working Paper* https://www.usitc.gov/publications/332/EC200809A.pdf (2008).
11. Headey, D. Rethinking the global food crisis: the role of trade shocks. *Food Policy* **36**, 136–146 (2011).
12. Fellmann, T., Hélaine, S. & Nekhay, O. Harvest failures, temporary export restrictions and global food security: the example of limited grain exports from russia, ukraine and kazakhstan. *Food Security* **6**, 727–742 (2014).
13. Anderson, K. & Nelgen, S. Trade barrier volatility and agricultural price stabilization. *World Dev.* **40**, 36–48 (2012).
14. Food & Organization, A. Faostat [data file] (2016). Retrieved from http://www.fao.org/faostat/en/#data/TM.
15. Weisstein, E. W. Simple directed graph (2015). Retrieved from http://mathworld.wolfram.com/SimpleDirectedGraph.html.
16. Nations, U. Economic commission for europe (2015) un/locode country names iso 3166-1. Retrieved from http://www.unece.org/cefact/locode/countries.html.
17. Scholz, F. W. & Stephens, M. A. K-sample anderson-darling tests. *J. Am. Stat. Assoc.* **82**, 918–924 (1987).
18. Garlaschelli, D. & Loffredo, M. I. Fitness-dependent topological properties of the world trade web. *Phys. Rev. Lett.* **93**, 188701 (2004).
19. Garlaschelli, D., Di Matteo, T., Aste, T., Caldarelli, G. & Loffredo, M. I. Interplay between topology and dynamics in the world trade web. *Eur. Phys. J. B* **57**, 159–164 (2007).
20. Albert, R., Jeong, H. & Barabási, A.-L. Error and attack tolerance of complex networks. *Nature* **406**, 378–382 (2000).
21. Rautureau, S., Dufour, B. & Durand, B. Targeted surveillance of cattle trade using social network analysis tools. *Rev. Epid. San. Anim.* **59/60**, 58–60 (2011).
22. Li, C., Zhao, H. & Zhang, X. A local-clustered evolving network model. *ICIC-EL* **2**, 193–199 (2008).
23. Ji, Q., Zhang, H. & Fan, Y. Identification of global oil trade patterns: an empirical research based on complex network theory. *Energ. Convers. Manage.* **85**, 856–865 (2014).
24. Iyer, S., Killingback, T., Sundaram, B. & Wang, Z. Attack robustness and centrality of complex networks. *PLoS ONE* **8**, e59613; 10.1371/journal.pone.0059613 (2013).

25. Holme, P., Kim, B. J., Yoon, C. N. & Han, S. K. Attack vulnerability of complex networks. *Phys. Rev. E* **65**, 056109 (2002).

26. Gephart, J. A., Rovenskaya, E., Dieckmann, U., Pace, M. L. & Brännström, Å. Vulnerability to shocks in the global seafood trade network. *Environ. Res. Lett.* **11**, 035008 (2016).

27. Thompson, D. *et al.* Economic costs of the foot and mouth disease outbreak in the united kingdom in 2001. *Rev. Sci. Tech. Off. Int. Epiz.* **21**, 675–685 (2002).

28. Sharma, R. Food export restrictions: review of the 2007-2010 experience and considerations for disciplining restrictive measures. *FAO Commodity and Trade Policy Research Working Papers* http://www.fao.org/fileadmin/templates/est/PUBLICATIONS/Comm_Working_Papers/EST-WP32.pdf (2011).

29. Gulati, A. & Saini, S. India's political economy responses to the global food price shock of 2007–08. *WIDER Working Papers 2015* (2015).

30. Jamakovic, A. & Uhlig, S. On the relationships between topological measures in real-world networks. *Netw. Heterog. Media* **3**, 345 (2008).

31. Barabási, A.-L. *Network science*. 59, 64, 236-237 (Cambridge University Press, 2016).

32. Newman, M. *Networks: an introduction*. 134 (Oxford University Press, 2010).
